# Screening and functional characterization of salt-tolerant NAC gene family members in *Medicago sativa* L

**DOI:** 10.3389/fpls.2025.1461735

**Published:** 2025-04-01

**Authors:** Zhiguang Li, Qianqian Yu, Yue Ma, Fuhong Miao, Lichao Ma, Shuo Li, Huajie Zhang, Zeng-Yu Wang, Guofeng Yang, Kunlong Su

**Affiliations:** ^1^ Key Laboratory of National Forestry and Grassland Administration on Grassland Resources and Ecology in the Yellow River Delta, College of Grassland Science, Qingdao Agricultural University, Qingdao, China; ^2^ Academy of Dongying Efficient Agricultural Technology and Industry on Saline and Alkaline Land in Collaboration with Qingdao Agricultural University, Dongying, China; ^3^ Weihai Animal Epidemic Disease Prevention and Control Center, Weihai, China; ^4^ Weihai Academy of Agricultural Sciences, Weihai, China

**Keywords:** alfalfa, NAc, salt tolerance, phylogenetic analysis, MsNAC40

## Abstract

**Introduction:**

Alfalfa is the most widely cultivated high-quality perennial leguminous forage crop in the world. In China, saline-alkali land represents an important yet underutilized land resource. Cultivating salt-tolerant alfalfa varieties is crucial for the effective development and utilization of saline-alkali soils and for promoting the sustainable growth of grassland-livestock farming in these regions. The *NAC* (*NAM, ATAF*, and *CUC*) family of transcription factors plays a key role in regulating gene expression in response to various abiotic stresses, such as drought, salinity and extreme temperatures, thereby enhancing plant stress tolerance.

**Methods:**

This study evaluated the structure and evolutionary relationship of the members of the NAC-like transcription factor family in alfalfa using bioinformatics. We identified 114 members of the *NAC* gene family in the Zhongmu No.1 genome and classified them into 13 subclasses ranging from I to XIII. The bioinformatics analysis showed that subfamily V might be related to the response to salt stress. Gene expression analysis was conducted using RNA-seq and qRT-PCR, and *MsNAC40* from subfamily V was chosen for further investigation into salt tolerance.

**Results:**

*MsNAC40* gene had an open reading frame of 990 bp and encoded a protein containing 329 amino acids, with a molecular weight of 3.70 KDa and a conserved NAM structural domain. The protein was hydrophilic with no transmembrane structure.After treating both the *MsNAC40* overexpressing plants and the control group with 150 mmol/L NaCl for 15 days, physiological and biochemical measurements revealed that these plants had significantly greater height, net photosynthetic rate, stomatal conductance, and transpiration rate compared to the control group, while their conductivity was significantly lower. Additionally, the levels of abscisic acid in the roots and leaves, along with the activities of peroxidase, superoxide dismutase, and catalase in the leaves, were significantly higher in the overexpressing plants, whereas the malondialdehyde content was significantly lower. Moreover, the Na^+^ content in the overexpressing plants was significantly reduced, while the K^+^/Na^+^ ratio was significantly increased compared to the control group.

**Discussion:**

These results indicated that the *MsNAC40* gene improved the salt tolerance of *Pioneer Alfalfa SY4D*, but its potential mechanism of action still needs to be further explored.

## 1 Introduction

Salt-affected soils are a valuable land resource in China, covering approximately 10% of the country’s total area. Effectively developing and utilizing these soils could significantly advance sustainable grassland agriculture. However, the salinity present in these soils presents major challenges to agricultural development (Shao et al., 2019). In this context, enhancing the salt tolerance of alfalfa is particularly important. Alfalfa, one of the most important perennial legume crops worldwide, is known for its high-quality forage production. In China, it plays a crucial role in the grassland industry and has significantly contributed to economic growth. As demand for alfalfa continues to rise, especially in regions with salt-affected soils, the need to boost alfalfa production has become increasingly urgent (Wan et al., 2023). Improving alfalfa’s salt tolerance not only enables more effective use of these soils but also greatly aids in ecological restoration and land management in these areas.

Salt stress, a significant abiotic stressor, severely impacts plant growth and productivity by inducing osmotic stress and ion toxicity. leading to physiological drought and metabolic disruptions (Deinlein et al., 2014). To combat these challenges, plants have developed various adaptive mechanisms, including osmotic adjustment, maintenance of ion homeostasis, and management of oxidative stress (Zhang et al., 2022). Recent advancements in molecular biology and genetic engineering have furthered our understanding of the genetic mechanisms behind salt tolerance in alfalfa. These developments have facilitated the identification and manipulation of key genes, paving the way for enhanced stress resilience in this vital crop.

*NAC* transcription factors are one of the largest families of transcriptional regulators widely found in plants and have been shown to be involved in various plant growth and developmental processes and abiotic stress responses (Diao et al., 2020). The acronym *NAC* is derived from the names of three genes containing specific structural domains: NAM (no apical meristem), ATAF1/ATAF2 (Arabidopsis transcription ACtivation factor 1/2), and CUC2 (cup-shaped cotyledon 2). Several *NAC* genes have been identified in various plants, including *Arabidopsis thaliana* (117), rice (151), grapevine (79), citrus (26), grape (26), poplar (163), soybean (152), and tobacco (152) (Hu et al., 2010; Le et al., 2011; Nuruzzaman et al., 2010; Rushton et al., 2008) Previous studies have revealed that members of the *NAC* transcription factor family are extensively involved in the regulation of growth and developmental processes in different plants, including seed development, embryo development, stem tip meristem formation, stem fibre development, leaf senescence, and cell division (Duval et al., 2002; Guo et al., 2005; Kim et al., 2007, 2006; Ko et al., 2007; Sperotto et al., 2009). In addition, it has been shown that the *NAC* transcription factors can regulate plant responses to various biotic and abiotic stresses in different plants. For example, *ANAC019, ANAC055, and ANAC072* positively regulate drought tolerance, salt tolerance and abscisic acid content in *Arabidopsis* (Tran et al., 2004). Several other NAC-like transcription factors are also related to stress tolerance in *Arabidopsis*. For example, *ANAC083, ANAC041, ANAC054, and ANAC084* positively regulate seed germination under salt stress (Balazadeh et al., 2010), while *NAC1* positively regulates growth hormone and root development (Guo et al., 2005), and *ANAC019*, *ANAC042*, and *ANAC102* positively regulate cold stress, heat stress, and waterlogging, respectively (Christianson et al., 2009; Jensen et al., 2010; Shahnejat-Bushehri et al., 2017; Yuan et al., 2019). NAC-like TFs typically refer to transcription factors that share similar domains or functions with the NAC transcription factor family. While they possess similar NAM domains, they may differ slightly in evolution or function. NAC-like transcription factors have also been studied more extensively in maize, rice, and soybeans. In soybeans, *GmNAC11, GmSIN1*, and *GmNAC20* can improve the salt tolerance, while *GmNAC20* can also improve the cold tolerance of the plants (Hao et al., 2011; Li et al., 2019). In rice, the regulatory effects of NAC-like transcription factors, such as *OsNAC4*, *OsNAC5*, *OsNAC6*, and *OsNAC10*, on abiotic stresses increase stress tolerance (Hu et al., 2008; Jeong et al., 2010; Sperotto et al., 2009; Zheng et al., 2009). *ZMsNAC1* positively regulates low-temperature, high-salt, drought, and ABA stresses in maize (Lu et al., 2012).

In this experiment, we analysed the structure and evolutionary relationship of the members of the NAC-like transcription factor family in alfalfa using bioinformatics by screening and evaluating the relative expression of 15 candidate genes that were responsive to salt stress based on the transcriptome of the salinity-tolerant alfalfa. We also investigated the relative expression of these genes in various tissues of alfalfa at various time points under salt treatment. We transformed *MsNAC40* into alfalfa using an overexpression vector to obtain transgenic material overexpressing the *MsNAC40* gene. The positive transgenic lines overexpressing the *MsNAC40* gene were subjected to phenotypic, physiological, biochemical, and metabolic analyses to preliminarily investigate the function of the gene in salinity tolerance. The study identified candidate genes for salt tolerance in alfalfa and provides a theoretical basis for the selection and breeding of alfalfa varieties for salt tolerance.

## 2 Materials and methods

### 2.1 Identification of the *NAC* family members in alfalfa

The Zhongmu No.1 whole protein sequence, genome open reading frame, and genome annotation file were obtained from the alfalfa Zhongmu No.1 genome website (https://modms.lzu.edu.cn/) (Fang et al., 2024). The HiddenMarkovModel profile (E-value>e^−10^) of NAM (PF02365) (Wang et al., 2010) was downloaded from the Pfam website (https://pfam.xfam.org/) to identify the members of the *NAC* gene family in the alfalfa genome, and to collect and analyze the gene names, gene IDs, and gene annotations associated with each identified *NAC* transcription factor. Moreover, the gene name, gene ID, number of amino acids encoded, molecular weight, isoelectric point, instability coefficient, fat coefficient, and total average hydrophilicity physicochemical indexes of each *NAC* gene family transcription factor were also compiled and analysed.To ensure the accuracy of the selected genes, the predicted NAC protein sequences will be submitted to InterProScan (http://www.ebi.ac.uk/interpro/serach/sequence-serach),CDD (http://www.ncbi.nim.nih.gov/Structure/bwrpsb/bwrpsb.cgi), Pfam, and SMART (http://smart.embl-heidelberg.de/)for sequence calibration and verification of conserved domains. Subsequently, the isoelectric points and molecular weights of the NAC family protein members will be analyzed using the ExPASy (http://web.expasy.org/compute_pi/) website.

### 2.2 Conserved motifs and gene structure analysis of the alfalfa NAC gene family

The conserved domains of the alfalfa NAC gene family were searched in the NCBI database (https://www.ncbi.nlm.nih.gov/Structure/cdd/wrpsb.cgi) to further analyse the *Medicago sativa NACs* (*MsNACs*). A phylogenetic tree of alfalfa *NAC* gene family members was constructed via MEGA11.0 using the Neighbor-Joining (NJ) method with the bootstrap value set to 1000.

### 2.3 Chromosomal localisation and covariance analysis of the alfalfa *NAC* gene members

The chromosomal positions of alfalfa *NAC* gene members were screened based on the genome annotation information of Zhongmu No.1, and the TBtools software was used to map the chromosomal localisation of alfalfa *NAC* genes. *Medicago polymorpha*, *Arabidopsis thaliana*, and *Medicago sativa* genomes were subjected to covariance analysis, and the interspecies and intraspecies similar genes were analysed functionally.

### 2.4 Analysis of the cis-acting elements of the alfalfa *NAC* gene members

The cis-acting elements located 2000 bp upstream of the gene region of the alfalfa NAC gene family members were analysed based on the Zhongmu No.1 genome obtained from the PlantCARE website (http://bioinformatics.psb.ugent.be/webtools/plantcare/html/) (Lescot et al., 2002). The cis-acting elements were analysed using the TBtools (Chen et al., 2020), and the different types of cis-acting elements were visualized using a heat map generated using TBtools.

### 2.5 Screening and expression analysis of *MsNAC* transcription factors under salt and alkali stress in alfalfa

To identify *MsNAC* transcription factors with different expression levels and screen for *MsNAC* genes with large expression differences between salt stress and alkali stress, we used transcriptome data of alfalfa subjected to salt and alkali stresses, as reported by the College of Grassland Science of Qingdao Agricultural University. The transcriptome data were generated from 4-week-old alfalfa seedlings subjected to salt, alkali, and salt-alkali mixed treatments. The treatments were divided into the following seven groups: Group A (control group), Group B (100 mmol/L NaCl solution), Group C (100 mmol/L NaHCO_3_ solution), Group D (90 mmol/L NaCl+10 mmol/L NaHCO_3_ solution), Group E (80 mmol/L NaCl+20 mmol/L NaHCO_3_ solution), Group F (70 mmol/L NaCl +30 mmol/L NaHCO_3_ solution), and Group G (60 mmol/L NaCl+40 mmol/L NaHCO_3_ solution). Samples were taken at days 1 and 6 for transcriptome sequencing. A heat map showing the expression of *MsNAC* genes was generated using TBtools, and **Table 1** presents the list of primers and their sequences.

**Table 1:** List of primers and their sequences.

Primername	Primersequence
Actin-F	ACTGGAATGGTGAAGGCTGG
Actin-R	TGACAATACCGTGCTCAATGG
qMsNAC4-F	TCATTACTTTTTATTTGC
qMsNAC4-R	ATCTCTTTATCTTTTCCA
qMsNAC30-F	TTGGAAAGCAACTGGAAA
qMsNAC30-R	CACGAGGGCTAAAGAAAT
qMsNAC29-F	AAGTTACCACCCTGTTTT
qMsNAC29-R	GTCTCCTCCCCGTTTTTG
qMsNAC40-F	TTCCAGAGAGAGATCCTC
qMsNAC40-R	CTCACCAAAATTCGCCTT
qMsNAC39-F	CACCTGGTTTCAGATTCT
qMsNAC39-R	CCCTCAACTTTTCTTTTT
qMsNAC50-F	AGCTTGATGTTATTCCAG
qMsNAC50-R	AATCTTTCTCTCTTTTCC
qMsNAC51-F	GGACACAAAATAGAATGA
qMsNAC51-R	CTAGAGGAAGAAGCAGAA
qMsNAC52-F	TGGGTTTGTCTTCTCTCC
qMsNAC52-R	GATTTATTTTCCTTCACT
qMsNAC85-F	TGGCAAGACCAAGTTTTT
qMsNAC85-R	GGTATGATGCTAGGATGA
qMsNAC79-F	ATTCTCCTCAGCTCTGTG
qMsNAC79-R	CTTTCTGCCTGCTCTCTT
qMsNAC70-F	TAAGGTCTTCTCTTTCCC
qMsNAC70-R	AACCAGTTGCTTTCCAGT
qMsNAC77-F	GATTGCCTCCTGGTTTTT
qMsNAC77-R	TGGCTTCCTTGCTGCTGA
qMsNAC78-F	ACAACAACAAGGAGAAAG
qMsNAC78-R	AGGTAATGAAATGGAAAT
qMsNAC108-F	TGGACACAGCCAAGACAG
qMsNAC108-R	GGGACACCAACAACAGCA
qMsNAC113-F	TGCTTCACACTTTTTCCA
qMsNAC113-R	GCTTTTCCTCCACTCTCC

### 2.6 Analysis of expression patterns of candidate MsNAC genes under salt treatment in root and leaf tissues

RNA was extracted using the Vazyme kit from the 2nd and 3rd leaves of the stem apical part and the 3 cm region of the root tips of 4-week-old hydroponic seedlings of Zhongmu No.1 sampled at 0h, 12h, 24h, and 48h after 50 mmol/L NaCl, 100 mmol/L NaCl, and 150 mmol/L NaCl salt treatment, as well as those subjected to double distilled water (ddH2O) incubation (control). The treatment procedures are detailed in **Table 2**. The RNA samples were reverse transcribed into cDNA for real-time fluorescence quantitative PCR analysis. The reaction conditions were as follows: *MsAction* was used as the internal control gene, and *MsNAC40* was the target gene. The relative expression levels of the genes in each group were determined using the *Ct* method, and the expression levels of the genes were calculated using Excel. The variability of the treatment groups was calculated using IBM SPSS Statistics25 software.

**Table 2:** List of Salt stress treatment methods and sampling sites.

Number	Salt treatment method	Sampling location
CK1	ddH2O	Root tip 2 to 3 cm
A1	50 mmol/L NaCl	Root tip 2 to 3 cm
B1	100 mmol/L NaCl	Root tip 2 to 3 cm
C1	150 mmol/L NaCl	Root tip 2 to 3 cm
CK2	ddH2O	The second and third leaves at the top of the stem
A2	50 mmol/L NaCl	The second and third leaves at the top of the stem
B2	100 mmol/L NaCl	The second and third leaves at the top of the stem
C2	150 mmol/L NaCl	The second and third leaves at the top of the stem

### 2.7 Cloning and protein structure analysis of the *MsNAC40* gene

The *MsNAC40* protein sequences were uploaded to ExPASy protparam (https://www.expasy.org/resources/protparam), ExPASy protscale (https://www.expasy.org/resources/protscale), and NetPhos3.1 (https://services.healthtech.dtu.dk/services/NetPhos-3.1/) platforms to analyse the primary structure, hydrophilicity and the phosphorylation sites of the *MsNAC40* proteins, respectively. Bio Lib (https://dtu.biolib.com/DeepTMHMM) was used for phosphorylation site mapping of the *MsNAC40* proteins, while TMHMM2.0 (https://services.healthtech.dtu.dk/services/TMHMM-2.0/) was used to predict the transmembrane helical structure of the *MsNAC40* proteins. Moreover, SWISS-MODEL (https://swissmodel.expasy.org/) was used to predict the tertiary structures of the *MsNAC40* proteins.

### 2.8 Tissue-specific expression of *MsNAC40*

Alfalfa SY4D was cultured to the early flowering stage, after which RNA was extracted from the root, stem, leaf, flower, and branch tissues of the plants. The RNA samples were reverse transcribed into cDNA, and real-time fluorescence-based quantitative PCR experiments were conducted, with each sample being repeated three times. The sample data were normalized based on the internal control MsAction, and the relative expression levels of the genes in each group were determined using the Ct method. The expression levels of the genes were calculated using Excel, and the IBM SPSS Statistics25 software was used to calculate the variability of each treatment group.

### 2.9 Identification of positive seedlings and expression analysis of positive transgenic plants

The 3301MsNAC40-F/R primers were designed with Nco1 and Pml1 enzymatic cleavage sites (**Table 3**) to obtain the target gene fragment with homologous arms for ligation into the linear vector pCAMIBA3301 digested with Nco1 and Pml1 restriction enzymes. Colony PCR of the transformed E. coli cells detected no error, and the E. coli cells were transferred into Agrobacterium EHA105.

**Table 3:** List of primers.

Primer name	Primer sequence
MsNAC40-F	ATGGGAGTTCCAGAGAGAGATCCTC
MsNAC40-R	TTAATGACCCGAATACCCAAACC
M13F	TGTAAAACGACGGCCAGT
M13R	CAGGAAACAGCTATGACC
Actin-F	ACTGGAATGGTGAAGGCTGG
Actin-R	TGACAATACCGTGCTCAATGG
qMsNAC40-F	TTCCAGAGAGAGATCCTC
qMsNAC40-R	CTCACCAAAATTCGCCTT

The vector was constructed and transformed into Agrobacterium EHA105 cells, which were then used for the leaf disc transformation of young leaves of 4-weeks-old alfalfa SY4D. The transformed leaves were co-cultured in SH3a media for 20 h in the dark and then transferred into the selection medium (attachment). The formed calluses were cultured in the dark for 2 to 3 months and were transferred to the MSBK medium for about one week under the photoperiod cycle of 16 h light/8 h dark for 30 to 45 d. The green-sprouting calluses were transferred to SH9a medium (attachment) until the tissues regenerated into plantlets. The regenerated plantlets were then grown in the glasshouse.

Thereafter, DNA was extracted from the young leaves of the regenerated plants for PCR analysis using primers 3301JY-F/R and 31JY-F/R to confirm the presence of the transformed gene. The bands matching the length of the target fragment were sequenced and compared with the target sequence to their similarity. Moreover, RNA was extracted from the leaf tissues of the transgenic plants overexpressing the target gene and wild-type alfalfa SY4D and reverse transcribed into cDNA. qPCR was performed to determine the expression level of the target gene in the transgenic alfalfa plants using qMsNAC40-F/R and Actin-F/R primers, and three overexpression plants with higher expression levels were selected.

### 2.10 Salt tolerance phenotype analysis of the overexpression alfalfa plants

Overexpression alfalfa SY4D plants with uniform growth were selected, and 9-cm cuttings were planted in nutrient soil in three pots. The pots were kept in the greenhouse for 30 d after daily treatment with 100ml of half-strength (1/2) Hoagland’s nutrient solution containing 150 mmol/L NaCl for 15 days. Thereafter, the absolute height from the ground to the highest point of the plant was measured in triplicate using a straightedge, and the three measurements were averaged. Fresh weight was measured by cutting the above-ground portion of the plant flush from the ground with scissors. The measurement was conducted in triplicate, and the three measurements were averaged.

### 2.11 Analysis of the physiological indicators of salt tolerance in alfalfa plants overexpressing the target gene

The plants were treated as described in section 2.10, and the apical 2nd and 3rd leaves were sampled at 10:30-11:00 a.m. to determine photosynthetic indexes, including net photosynthetic rate, stomatal conductance, and transpiration rate. Ten leaves were sampled from each treatment, and the average value was determined. Furthermore, 0.3g of fresh leaves were weighed and cut into small sections (1.5 cm) to determine the initial conductivity E1. The leaves were then incubated in boiling water for 20 min to cool down to determine the second conductivity E2. The measurements were repeated 3 times, and the relative conductivity was then calculated as (E2-E1)/E2.

### 2.12 Analysis of the biochemical indicators of salt tolerance and the contents of K^+^ and Na^+^ in root and leaf tissues of alfalfa plants overexpressing the target gene

The plants were treated as described in section 2.10. Appropriate amounts of leaves were sampled for measuring the proline, malondialdehyde, peroxidase, superoxide dismutase, and catalase contents using the Solepol activity test kit. After 12h of treatment with 100ml of 1/2 Hoagland nutrient solution containing 150 mmol/L NaCl, the leaves and roots were collected for measuring the abscisic acid contents using the Solepol Abscisic Acid Activity Test Kit.

The plants were treated as described in section 2.10, and the root tip tissues were sampled and oven-dried for 1h at 105°C, followed by 24h at 80°C. Thereafter, the samples were ground to powder and weighed (indicate the amount weighed here) for a 2h digestion using 1mL of HNO_3_ and 1 mL of H_2_O_2_. The supernatant was collected and left standing overnight. The k^+^ and Na^+^ standard solutions were diluted to 0, 5, 10, 20, 30, 40, and 50 µg/mL with ultrapure water, and the standard curve was plotted. The samples were diluted 10 times with ultrapure water, and the concentrations of k^+^ and Na^+^ were determined using an M425 flame spectrophotometer. The final contents of k^+^ and Na^+^ in alfalfa roots were calculated as [C (measured concentration) × V (volume of measured liquid) × K (fractionation times)]/m (mass of dried sample).

## 3 Results

### 3.1 Identification of *NAC* family members in alfalfa

**Figure 1:**
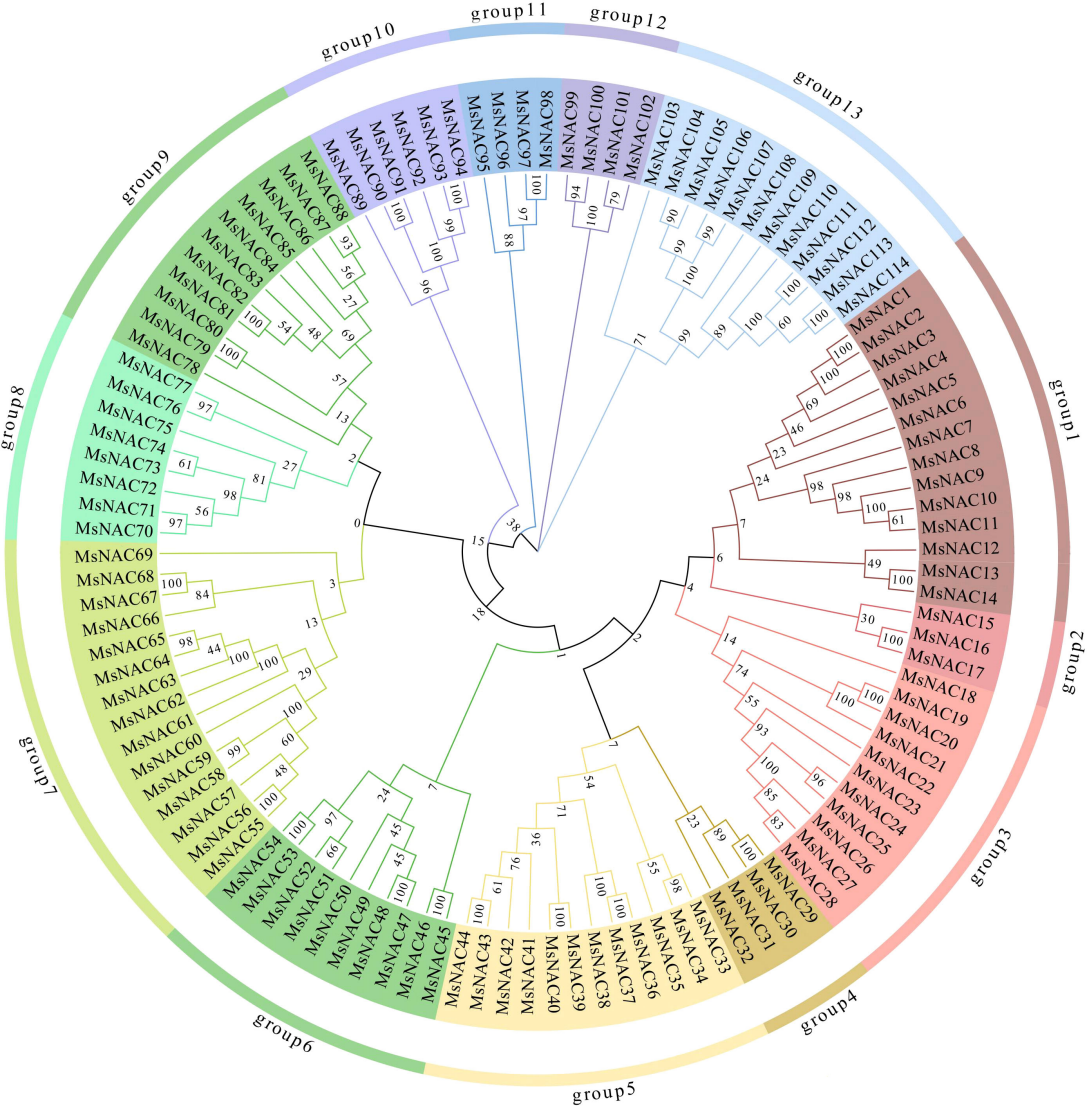
Evolutionary tree of Alfalfa *NAC* gene family.

We screened 114 *NAC* genes from the Zhongmu No.1 genome, as shown in **Table 1**. The statistical results showed that the number of amino acids, the molecular weight, the isoelectric point, the instability coefficient, and fat coefficients of the alfalfa *NAC* family members ranged from 64 to 1094aa, 7.24 to 124.7 kDa, 4.09 to 9.84, 21.68 to 62.88, and 51.61 to 89.88, respectively (**Supplementary Table S1**). According to the prediction results, all *NAC* family members were hydrophilic proteins. Moreover, we analysed the results of the subfamily classification of Medicago truncatula, A. thaliana, and Soybean and found that the alfalfa *NAC* family classifies into 13 subfamilies, named subfamilies I to XIII (Le et al., 2011; Ling et al., 2017a, 2017; Ooka et al., 2003). The number of gene members in each I-XIII subfamilies was 14, 3, 11, 4, 12, 10, 15, 8, 11, 6, 4, 4, and 12, in that order, and the genes were named according to their order in the evolutionary tree, from *MsNAC1* to *MsNAC114* genes **Figure 1**.

### 3.2 Conserved motifs and gene structure analysis of the alfalfa *NAC* gene family

**Figure 2:**
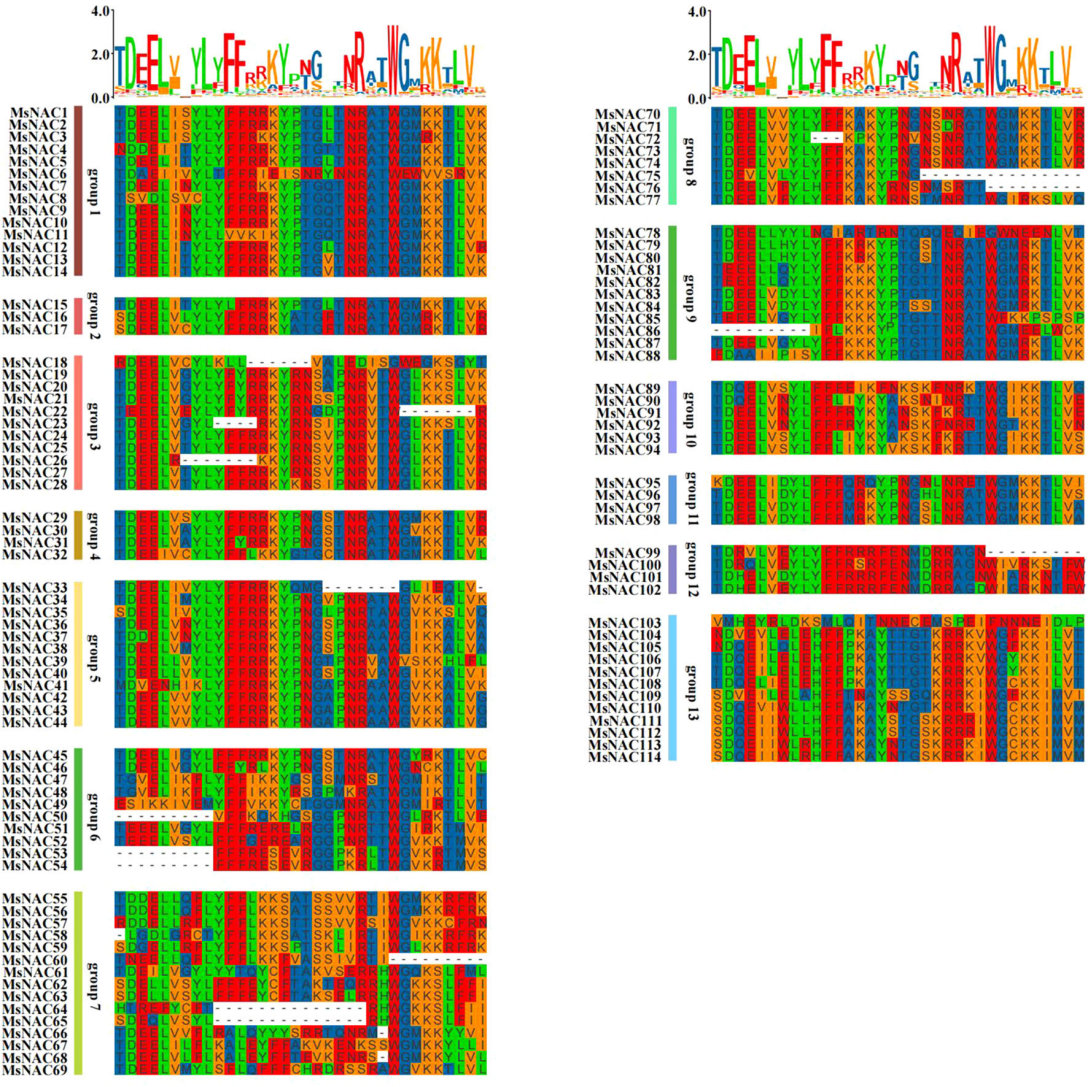
Analysis of conserved domain of Alfalfa *NAC* gene family.

As shown in **Figure 2**, most of the conserved domains of the *NAC* genes matched the NAM sequences. The amino acid deletions were classified into three types: deletions at the beginning of *MsNAC50*, *MsNAC53*, *MsNAC54*, and *MsNAC86*, deletions at the end of *MsNAC22*, *MsNAC60*, *MsNAC75*, *MsNAC76*, and *MsNAC99*, and deletions at the middle of *MsNAC26*, *MsNAC64*, and *MsNAC65*.

**Figure 3:**
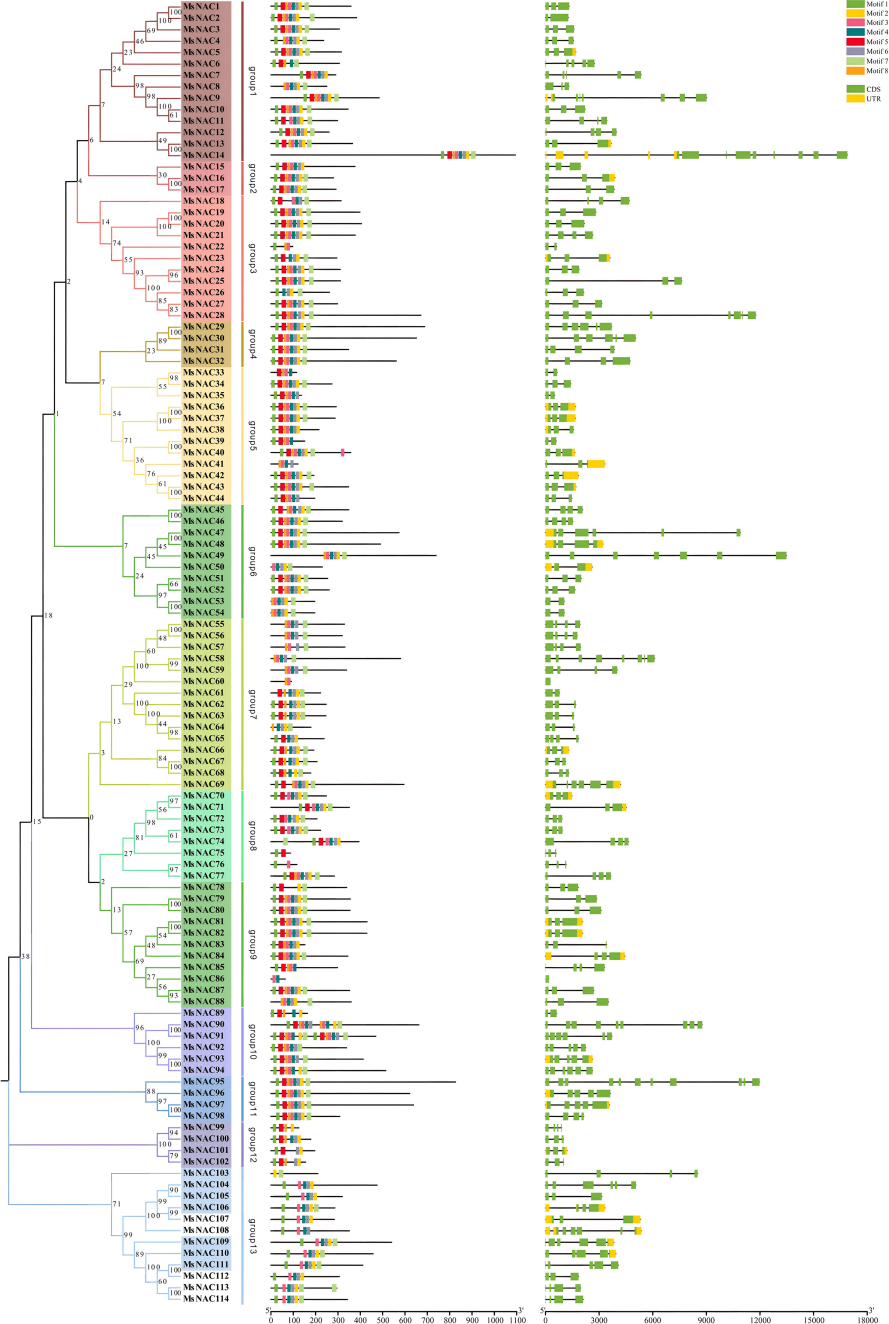
Alfalfa *NAC* gene family Evolutionary tree, motif and gene structure analysis.

The 114 NAC-like genes were subjected to motif analysis, and eight high-confidence motifs, named motif1-motif8, were selected for further analysis. It was found that most of the *NAC* gene family members contained these eight motifs, and the most abundant motif was motif 1, followed by motif 5, motif 8, motif 3, motif 4, motif 6, motif 2, and motif 7. The gene structure of the alfalfa *NAC* family members was analysed, and as shown in **Figure 3**, all alfalfa *NAC* family genes contained introns, and more genes contained more than five introns. Subfamilies V, VIII, and XII had relatively simple gene structures and high structural similarity within the subgroups.

### 3.3 Chromosomal localisation and covariance analysis of the alfalfa *NAC* gene members

**Figure 4:**
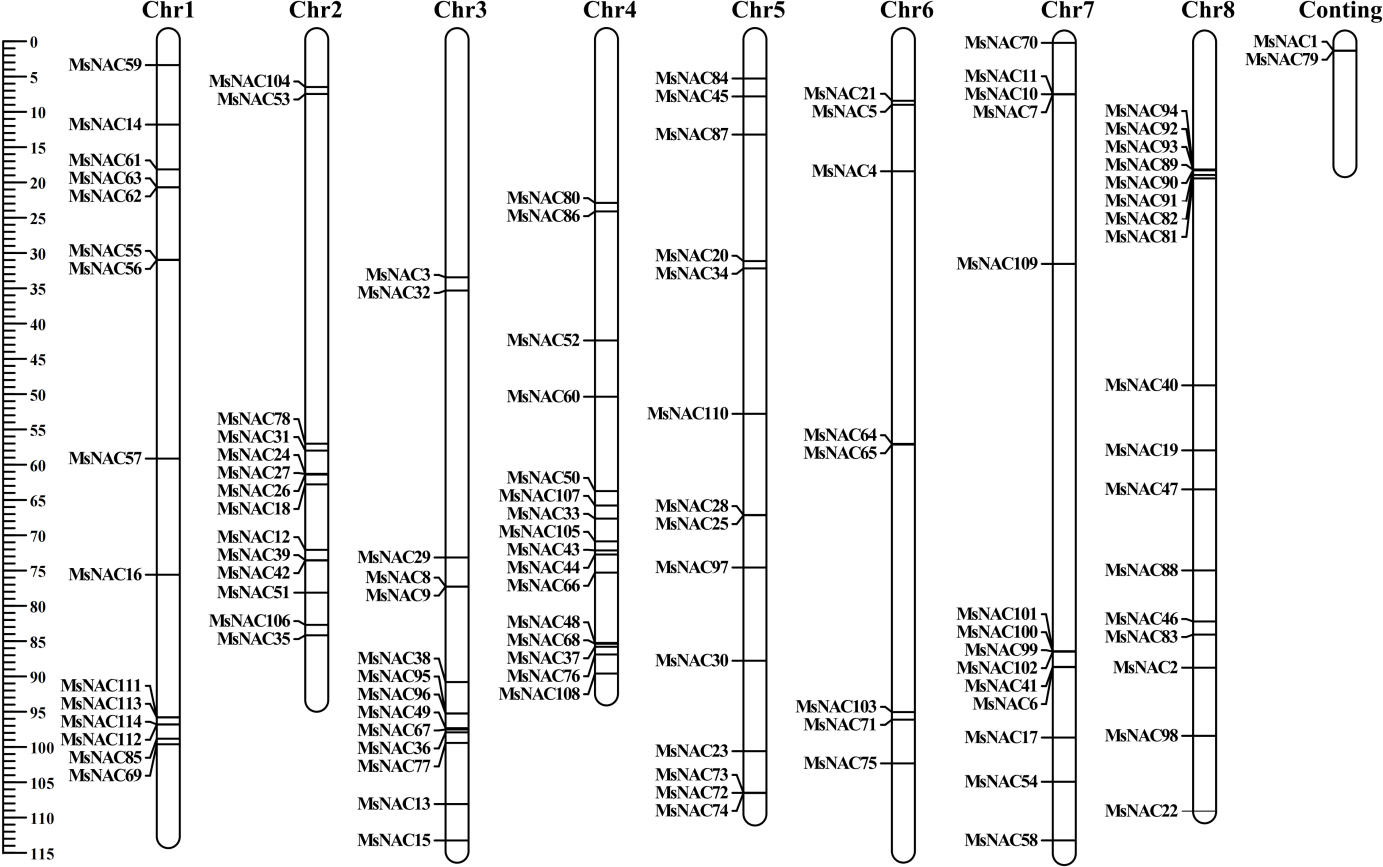
Chromosome mapping of Alfalfa *NAC* gene family.

**Figure 5:**
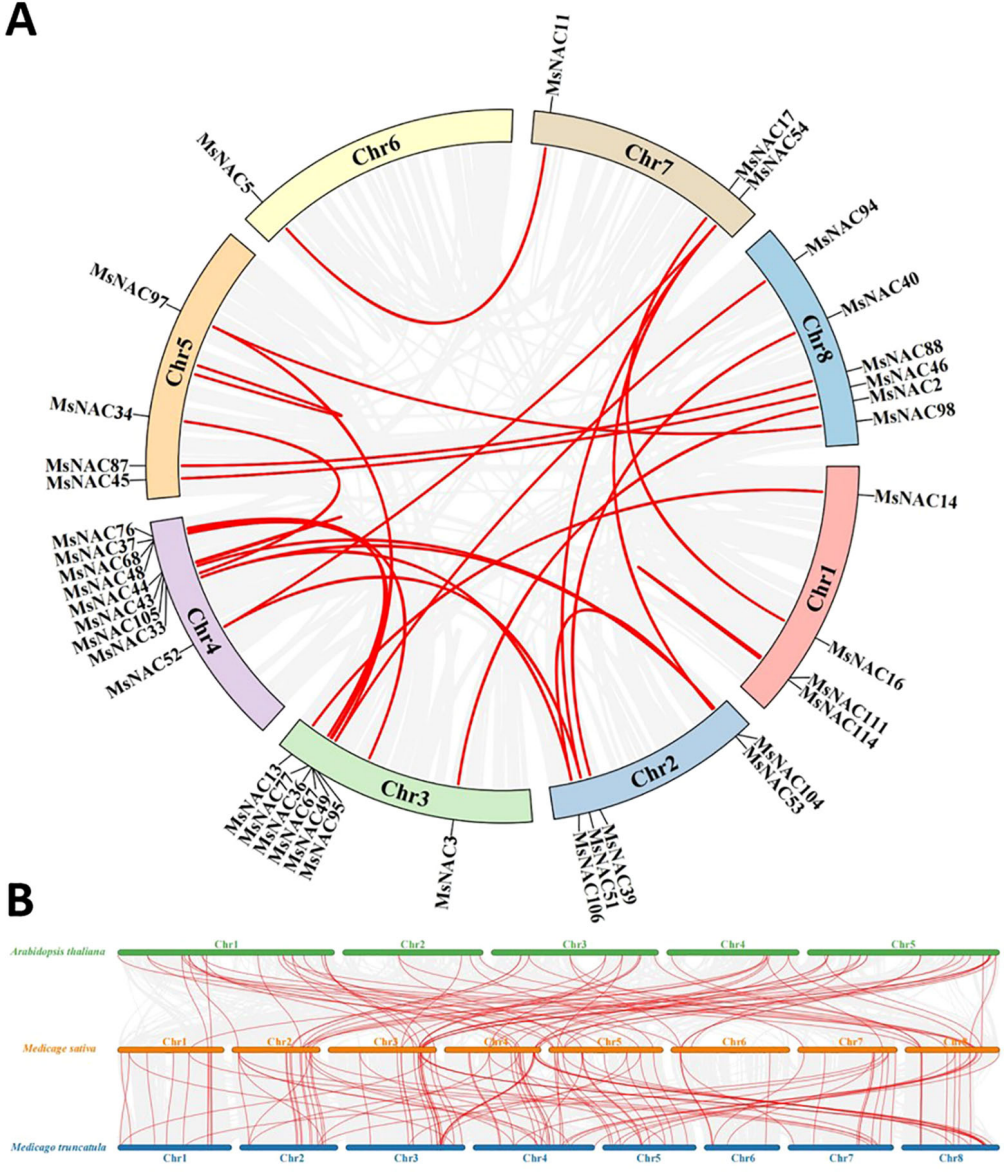
Collinearity analysis of Alfalfa *NAC* gene family. Red is collinear gene in gene family; **(A)** shows the collinearity of the *NAC* gene family, and **(B)** shows the collinearity analysis of Alfalfa with Arabidopsis and Tribulus. The gray line is the collinear block of Arabidopsis Tribulus and Alfalfa, and the red line is the *NAC* homologous gene pair.

As shown in **Figure 4**, the number of genes on chromosomes 1 through 8 were 15, 14, 14, 16, 14, 8, 14, and 17, respectively. Three pairs of tandem duplications (25/28, 43/44 and 111/114) and 21 pairs of segmental duplications (104/105, 104/106, 5/11, 14/13, 16/17, 3/2, 36/37, 39/40, 33/34, 45/46, 49/48, 51/52, 51/54, 53/54, 52/54, 67/68, 77/76, 87/88, 95/94, 95/97, and 97/98) were identified from the alfalfa *NAC* gene family via intraspecific covariance analysis (**Figure 5A**). We found that tandem duplications and fragmental duplications were mostly in the same subfamily, indicating high genetic similarity within the same subfamily. Moreover, the 21 pairs of fragmental duplications were mostly concentrated on chromosomes 2, 3, 4, 5, 7, and 8, and less on chromosomes 1 and 6. The frequent occurrence of fragmental duplications in chromosomes 3 and 4 might have been due to the recombination of chromosomes 3 and 4, indicating the dominant role of fragmentary duplications in promoting the evolution of the alfalfa *NAC* family.

The genome sequences of the widely studied model plant, A. thaliana, and the model legume plant, *Medicago sativa*, were used as templates to determine the functions of the identified alfalfa *NAC* genes. A total of 72 pairs of homologous genes were found in Medicago truncatula, and 117 pairs of homologous genes were found in alfalfa with thistles (Le et al., 2011; Ling et al., 2017b; Zhu et al., 2014). In this study, phylogenetic analysis of the alfalfa NAC gene family confirmed that alfalfa is a homotetraploid. The NAC genes in the Zhongmu No.1 genome were distributed across eight chromosomes, while in the Xinjiang Large-Leaf genome, they were distributed across 32 chromosomes, reflecting the fact that the Xinjiang Large-Leaf alfalfa genome consists of four haploid genome sets, whereas Zhongmu No.1 consists of only one. Given this fundamental genomic difference, direct comparisons of NAC gene family distribution between the two genomes may have limited significance.To further advance research on the NAC gene family in alfalfa, the **Supplementary Materials** of this paper provide a comparative analysis of NAC gene members between Zhongmu No.1 and Xinjiang Large-Leaf alfalfa. The observed gene distribution aligns with patterns seen in other polyploid species, such as cotton (Wu et al., 2013), and may reflect evolutionary pressures and functional adaptations of polyploid plants under abiotic stress conditions. Finally, based on the expression levels of candidate genes, MsNAC40 from subfamily V was selected as the focus for further research.

### 3.4 Analysis of the cis-acting elements of the alfalfa NAC gene members

**Figure 6:**
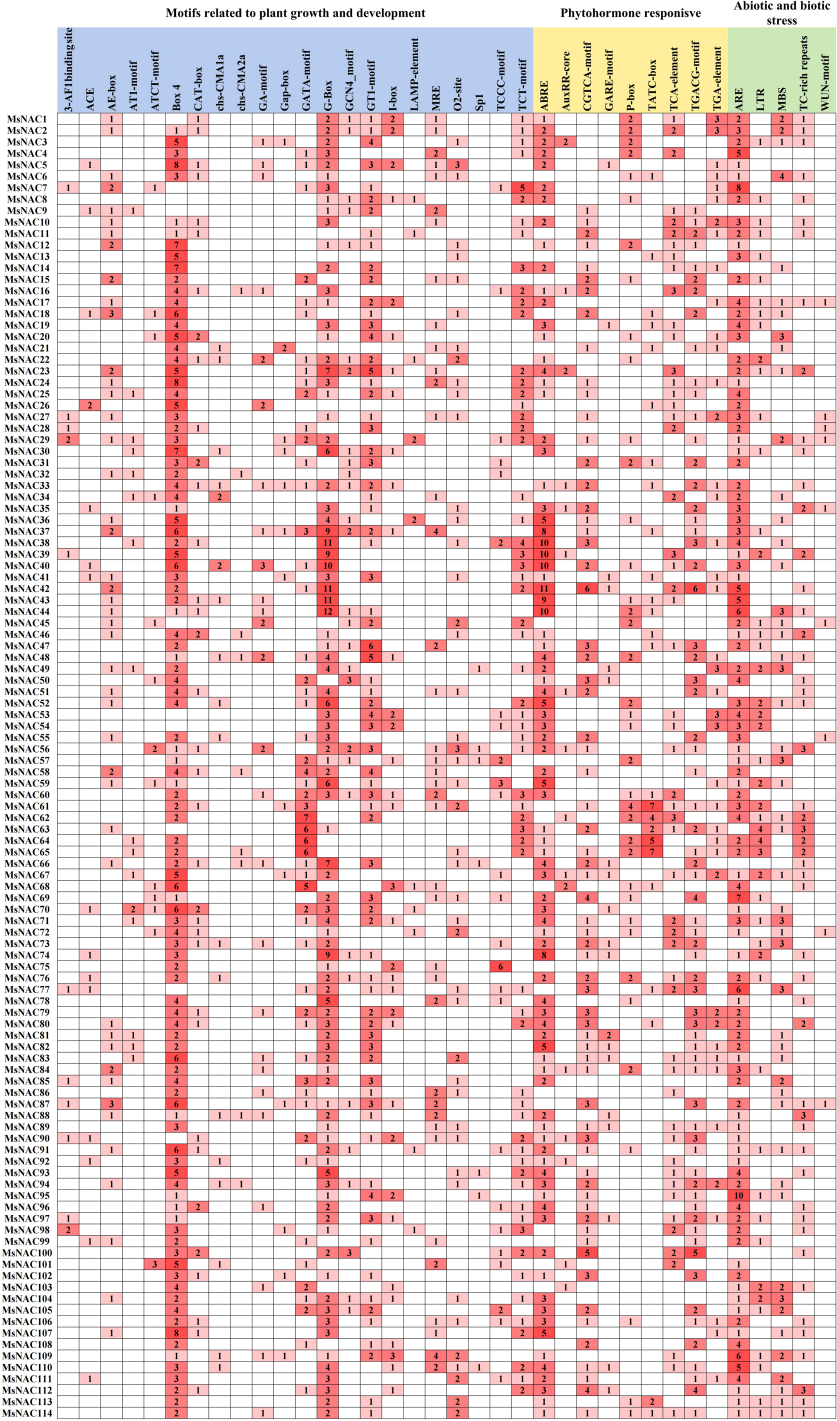
Analysis of promoter cis.acting elements of Alfalfa *NAC* gene family members. The grid colors and numbers represent the number of different cis.acting elements in the *MsNAC* gene and are presented as a heat map.

We analysed the sequence of the 2000 bp upstream of the promoter region of the alfalfa *NAC* genes. As shown in **Figure 6**, the cis-acting elements were mainly classified into three categories: (1) the plant growth and development category, which contained photosynthesis-related elements, such as G-box, Box4, GT1-motif, etc; (2) the plant hormone response category containing elements related to abscisic acid, gibberellin and other hormone responses, such as ABRE, P-box, etc; (3) the abiotic and biotic stress category containing anaerobic induction and other abiotic response-related elements, such as ARE, DRE, MBS, TC-richrepeats, etc. Through cluster analysis, we found that the *NAC* gene members in subfamily V contained more ABRE and ARE elements, suggesting that they may regulate the responses to abscisic acid and anaerobic stress. Moreover, subfamily V members contained many drought- and salt-stress-responsive elements, while those of subfamilies VI, VII, VIII, and XIII contained more LTR elements. Subfamily XIII members contained more MBS elements.

### 3.5 Screening and expression analysis of *MsNAC* transcription factors under salt and alkali stress in alfalfa

**Figure 7:**
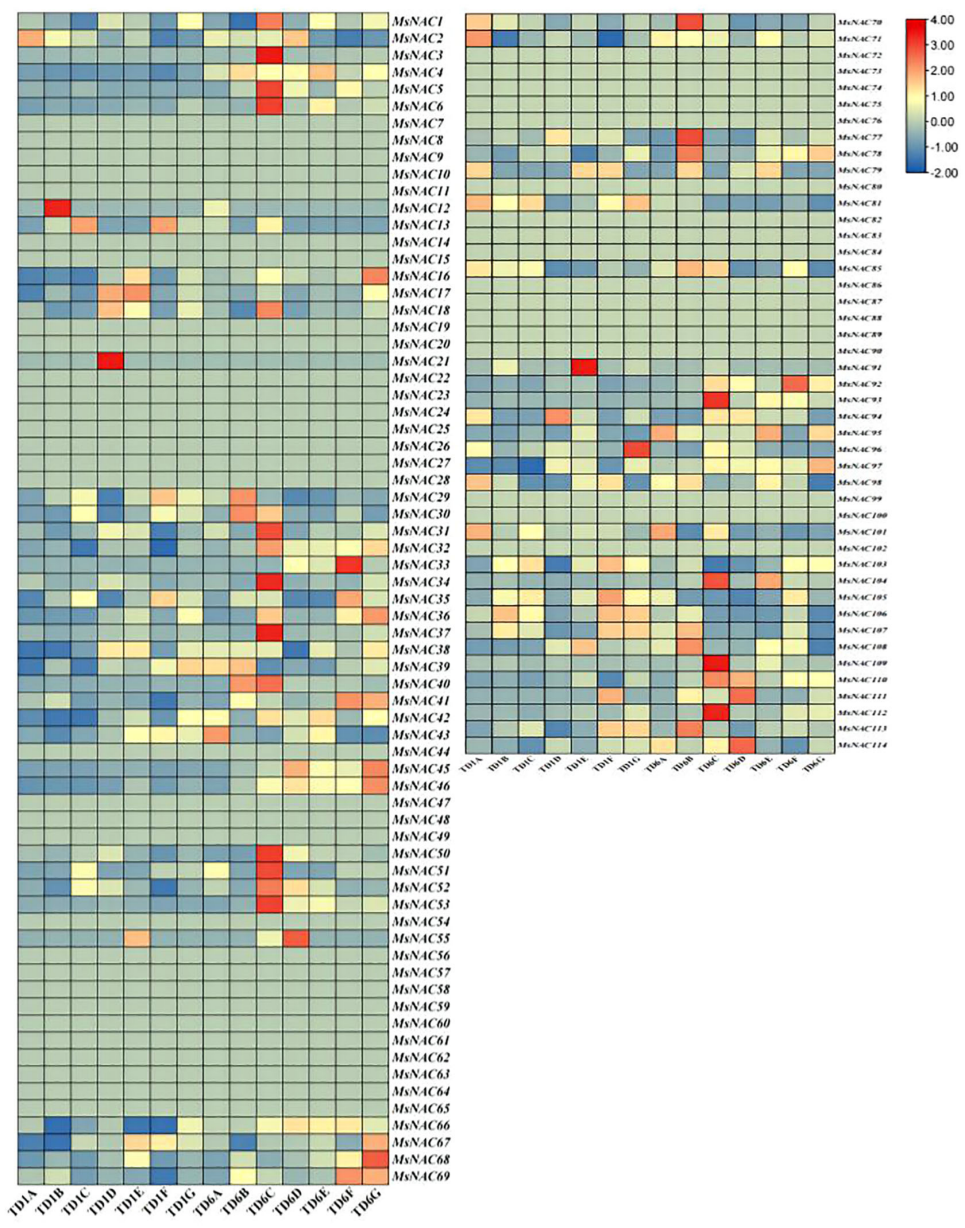
Relative expression calorimetry of Alfalfa *NAC* family gene under saline.alkali stress. The expression levels of *NAC* gene family members under different treatments at different time points were searched from the saline.alkali stress transcriptome. Blue and gray in the figure showed no matching data.The letters in each group are processing days and the last letter is each group.Group A is blank group and group B is 100 mmol/L NaCl solution, group C was 100 mmol/L NaHCO_3_ solution, group D was 90 mmol/L NaCl, 10 mmol/L NaHCO_3_ solution, group E was 80 mmol/L NaCl, 20 mmol/L NaHCO_3_ solution, group F was 70 mmol/L NaCl,30 mmol/L NaHCO3 solution,G group was 60 mmol/L NaCl, 40 mmol/L NaHCO_3_.

The transcriptomic data, which was obtained from the College of Grassland Agriculture at Qingdao Agricultural University, identified 74 *NAC* transcription factors with significantly different expression levels. As illustrated in **Figure 7**, sequence alignment was used to map these 74 *NAC* transcription factors to the categorized alfalfa *NAC* gene family, resulting in 74 of the 114 genes having corresponding data. The data were row normalised, after which 12 genes (4, 29, 30, 39, 40, 76,77, 78, 79, 85, 108, and 113) with large differences in their expression levels under salt stress and three genes (50, 51, and 52) with large differences in their expression levels under alkali stress and mixed saline and alkaline stress were screened.

### 3.6 Analysis of expression patterns of candidate MsNAC genes under salt treatment in root and leaf tissues

**Figure 8:**
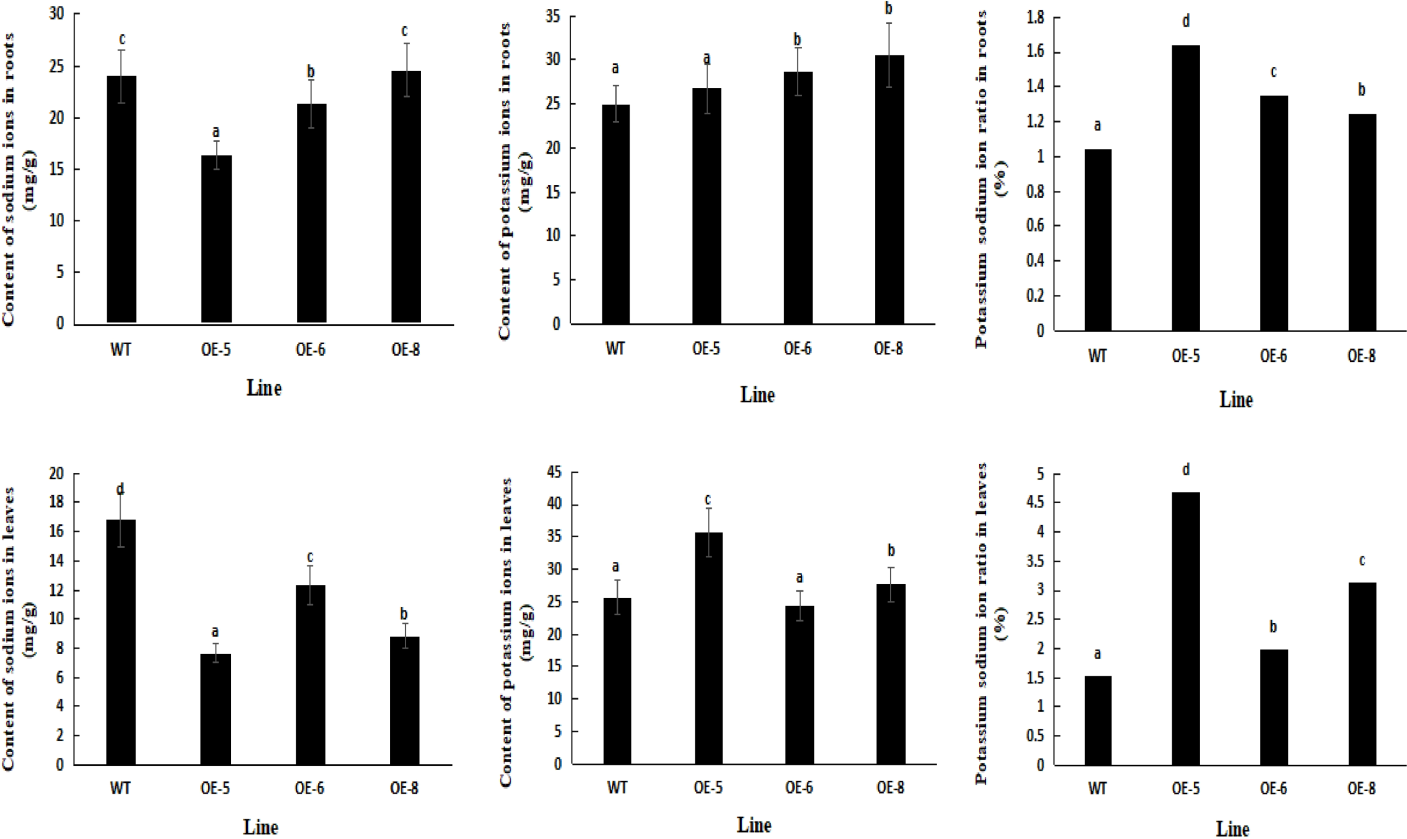
The expression levels of 15 candidate genes in root and leaf under different stress. The blue group is the blank control group, and the red group is 50 In the mmol/L NaCl salt treatment group, the gray was 100 mmol/L In the NaCl salt treatment group, yellow was 150 mmol/L NaCl salt treatment group.In bar charts, “abc” denotes the results of significance analysis.

We further analysed the expression levels of 15 candidate genes in root and leaf tissues under different concentrations of salt treatment. The results showed that the expression levels of seven genes (*MsNAC39*, *MsNAC40*, *MsNAC76*, *MsNAC77*, *MsNAC78*, *MsNAC85*, and *MsNAC108*) in the root and leaf tissues, expression levels of *MsNAC51* in the leaf tissues and the expression levels of *MsNAC79* in the root tissues increased under salt treatment (**Figure 8**). Moreover, the expression levels of *MsNAC40* and *MsNAC78* in the root and leaf tissues, the expression of *MsNAC77* in the leaf tissues, and the expression of *MsNAC79* in the root tissues increased with the increasing salt concentration. The highest fold change of the relative expression of *MsNAC40* in the root and leaf tissues was more than 8-fold. Thus, based on these results and those of transcriptomic data and cis-acting elements analysis, *MsNAC40* was selected for further analysis.

### 3.7 Cloning and protein structure analysis of the MsNAC40 gene

**Figure 9:**
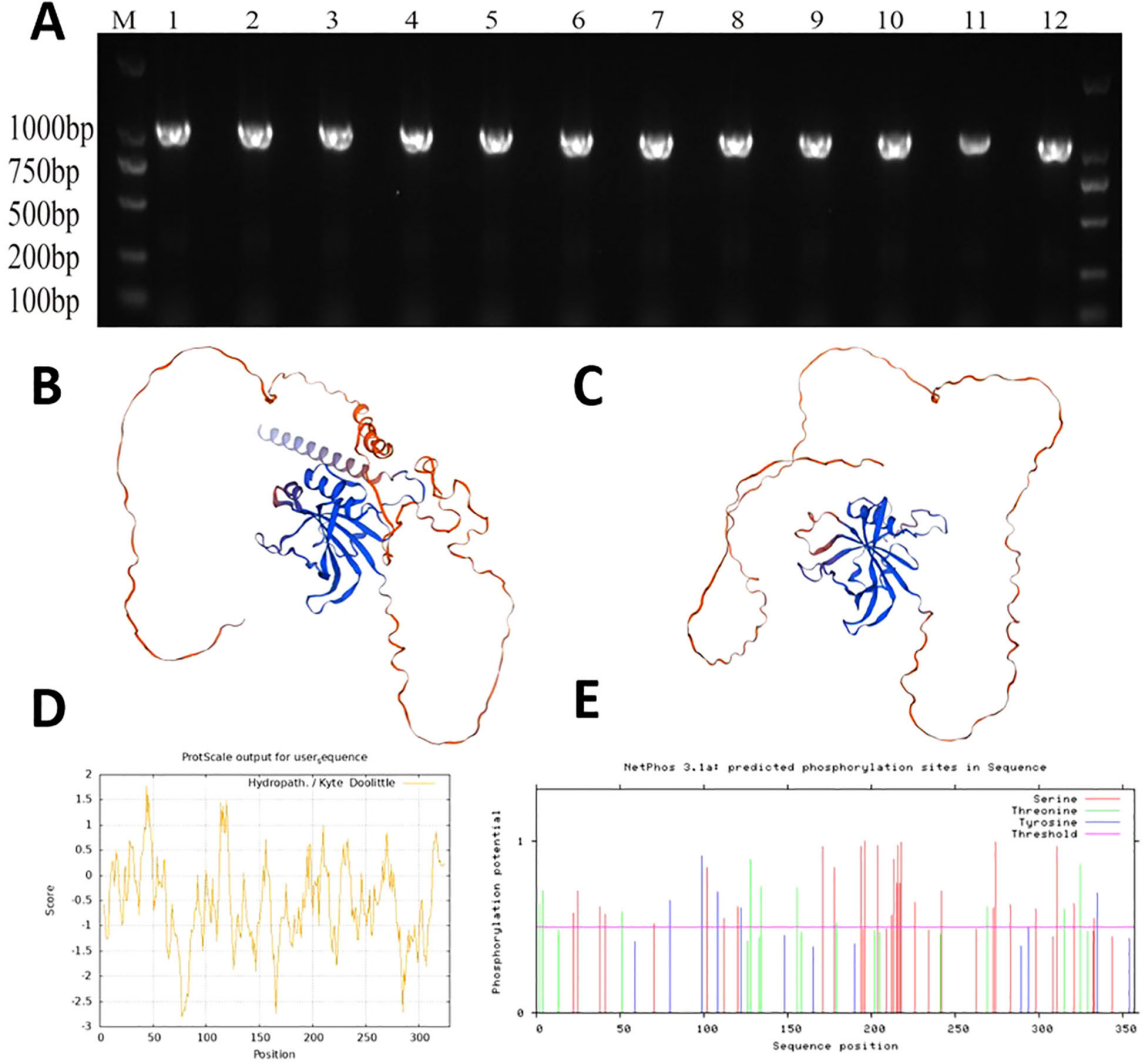
Analysis of *MsNAC40* Gene and Protein Structure and Function Prediction. **(A)** is the clone of *MsNAC40*, **(B)** is the predicted tertiary structure of *OsNAC1* protein, **(C)** is the predicted tertiary structure of *MsNAC40* protein, **(D)** is the predicted hydrophilicity of *MsNAC40*, and **(E)** is the predicted phosphorylation site of *MsNAC40*.

As shown in **Figure 9**, the *MsNAC40* gene was cloned from alfalfa SY4D, sequenced, and compared with the protein sequence encoded by Zhongmu No.1 *MsNAC40* (MsG0880044354.01.T01), The results showed that the cloned *MsNAC40* gene had 99% similarity with the Zhongmu No.1 *MsNAC40*. The length of the complete open reading frame of *MsNAC40* was 990bp, encoding 329 amino acids, and its protein structural domain was NAM (PF02365), belonging to the NAC-like gene family. *MsNAC40* was classified under the subfamily V in the alfalfa *NAC* family evolutionary tree.

Furthermore, the *MsNAC40* protein was 96.96% homologous to *MtNAC3* of T. terrestris alfalfa. The SWISS-MODEL results showed that the rice *NAC1* protein model 3ulx.1.B was the homologous model of *MsNAC40* protein, with a GMQE value of 0.69 and a similarity of 67.86%. As shown in **Figures 9B, C**, the NAM conserved domain overlapped the area of high prediction confidence, indicating that the functionally conserved NAM domains of the *MsNAC40* protein have high similarity with the rice *NAC1* protein model 3ulx. The physicochemical properties of *MsNAC40* protein were analysed using ExPASy (**Figures 9D, E**). We found that the number of encoding amino acids was 329, and the protein had a molecular weight of 3.70 KDa, an isoelectric point of 6.19, a total number of negatively charged amino acid residues (Asp+Glu) of 39, and a total number of positively charged amino acid residues (Arg+Lys) of 36. The hydrophilicity of the *MsNAC40* protein was analysed, and it was found that the 78th amino acid residue had the best hydrophilicity (-2.800), while the 45th amino acid had the best hydrophobicity (1.778). Additionally, there were more hydrophilic amino acid residues than hydrophobic ones, and the *MsNAC40* protein had no transmembrane structure.

Protein phosphorylation sites on the *MsNAC40* protein were predicted via the NetPhos website. As shown in **Figure 9E**, there were 40 phosphorylation sites on the *MsNAC40* protein, of which the serine, threonine, and tyrosine phosphorylation sites were 27, 8, and 5, respectively.

### 3.8 Tissue-specific expression of MsNAC40

**Figure 10:**
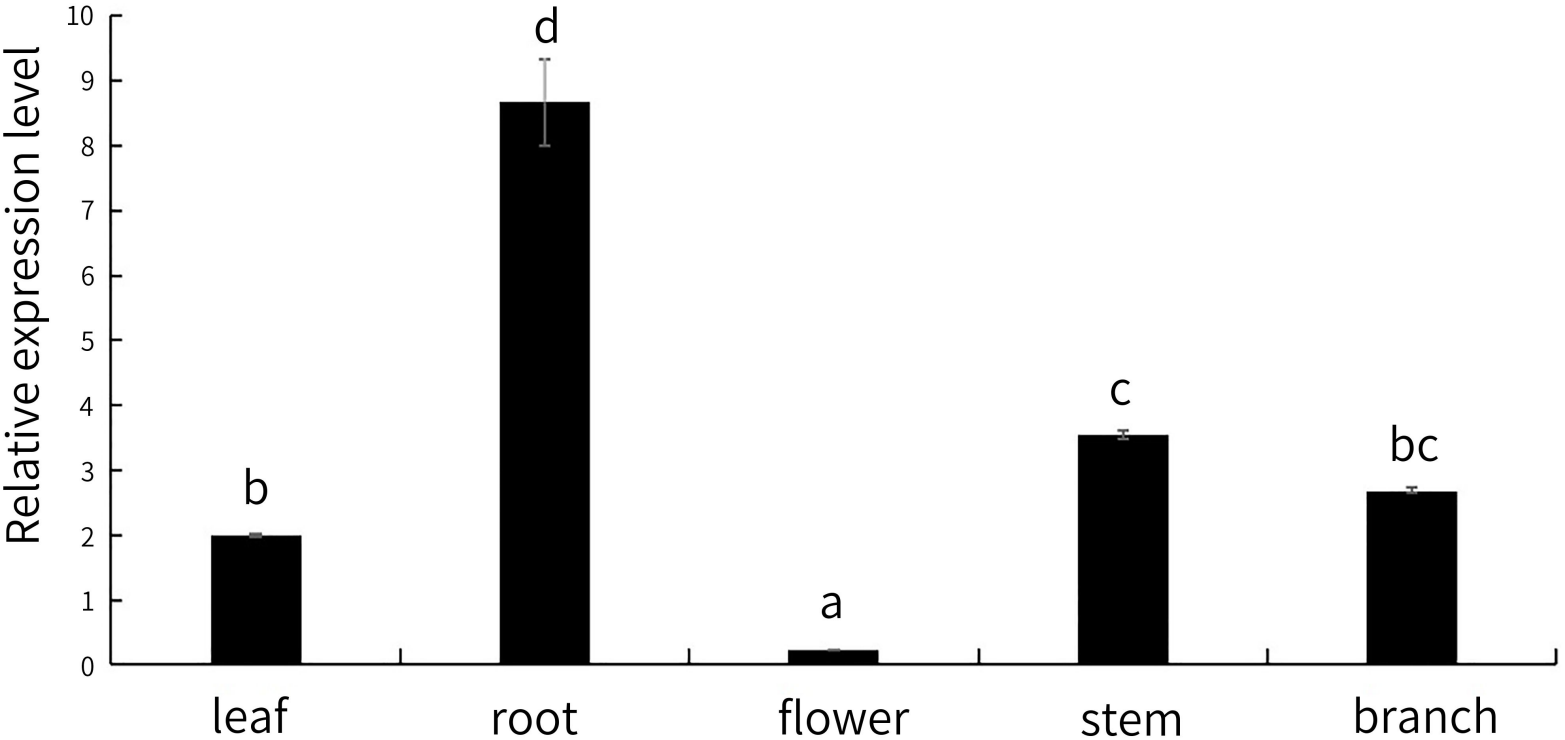
Expression levels of *MsNAC40* in different tissues. In bar charts, “abc” denotes the results of significance analysis.

We analysed the expression level of *MsNAC40* in the leaf, root, flower, stem, and branch tissues of 4-week-old alfalfa plants. As shown in **Figure 10**, the relative expression levels of the target gene in the different tissues were ordered as roots>stems> branches> leaves>flowers, indicating the relative expression of the gene in root tissues was significantly higher but significantly lower in flower tissues than in the other tissues. This suggests that *MsNAC40* may play a primary function in plant roots and a secondary function in stem, branch and leaf tissues.

### 3.9 Identification of positive seedlings and expression analysis of positive transgenic plants

As depicted in **Supplementary Figure S3**, through the operation of Agrobacterium-mediated leaf disc transformation, we found that 35 *MsNAC40*-overexpressing alfalfa seedlings were positive for the target gene (**Supplementary Figure S3A**). The first batch of alfalfa seedlings used for the identification of positive plants contained 1 to 7 lines, and sequencing results showed that the amplified gene from the seven lines was consistent with the target sequence (**Supplementary Figure S3B**), similar to those amplified from lines 8 to 35 (**Supplementary Figure S3C**).

**Figure 11:**
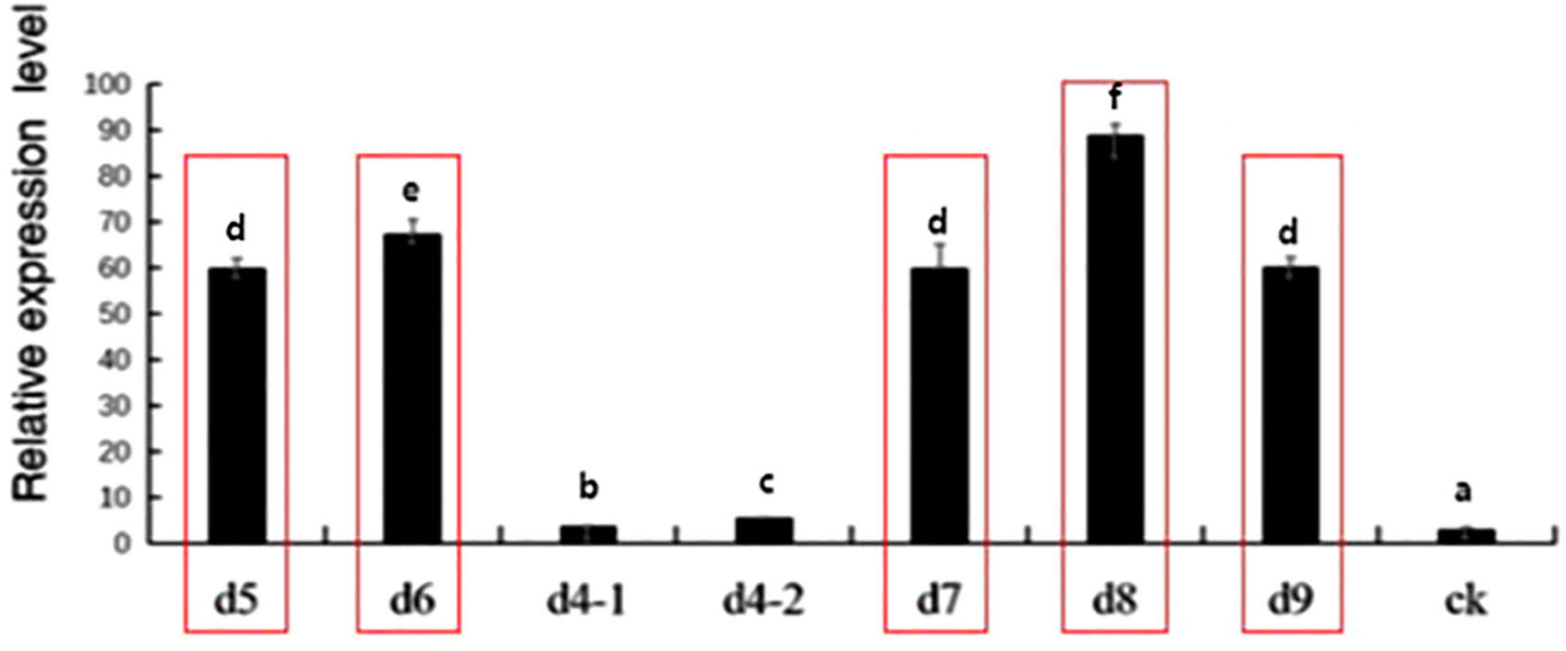
Expression level of *MsNAC40* in overexpressed positive plants. M is 1500bp DNA Marker, WT is pCAMIBA3301 vector with empty plasmid as the template, and 1 to 35 are the 35 *MsNAC40.*overexpressing seedlings positive for the target gene.In bar charts, “abc” denotes the results of significance analysis.

The qPCR experiments were also performed on the 35 overexpression lines, and the three lines with the highest expression were selected for subsequent analysis. As shown in **Figure 11**, lines d5, d6 and d8 had higher expression levels and were named L5, L6 and L8.

### 3.10 Salt tolerance phenotyping of the alfalfa plants overexpressing the target gene

**Figure 12:**
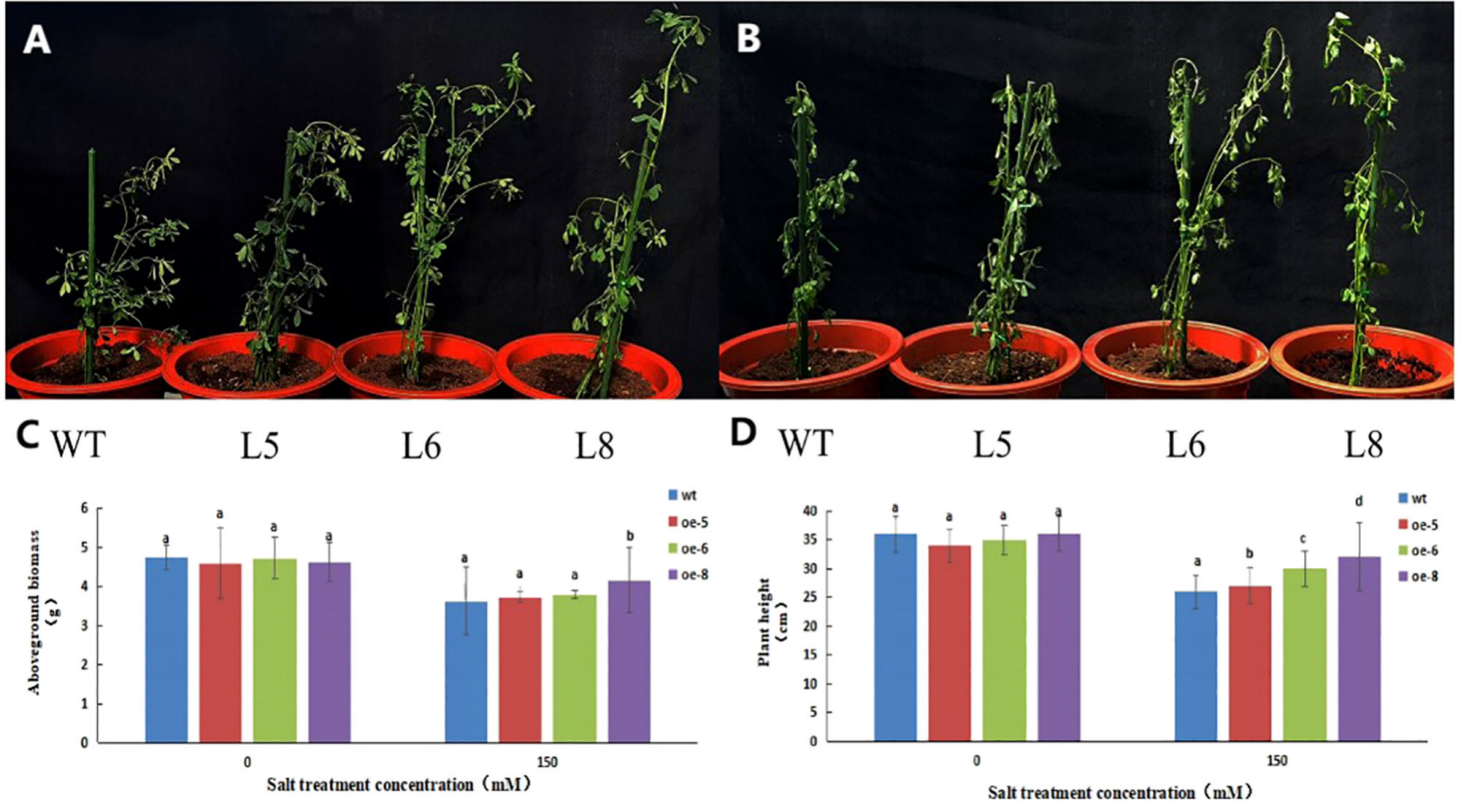
Phenotypic analysis of *MsNAC40* overexpressed plants under salt stress. **(A)** is the growth state under normal condition; **(B)** is the growth state after salt treatment for 15d; **(C)** is the fresh weight of the plant; **(D)** is the plant height.In bar charts, “abc” denotes the results of significance analysis.

As shown in **Figure 12**, there was no difference in the plant height and fresh weight between the control alfalfa plant SY4D and the transgenic lines under control conditions. However, after 15d of salt treatment, the fresh weight and plant height of line 8 was significantly higher than that of the control, and the plant height of lines 5 and 6 was significantly higher than that of the control. This indicated that *MsNAC40*-overexpressing alfalfa plants grew better under the 150 mmol/L NaCl treatment.

### 3.11 Analysis of the physiological indicators of salt tolerance in alfalfa plants overexpressing the target gene

**Figure 13:**
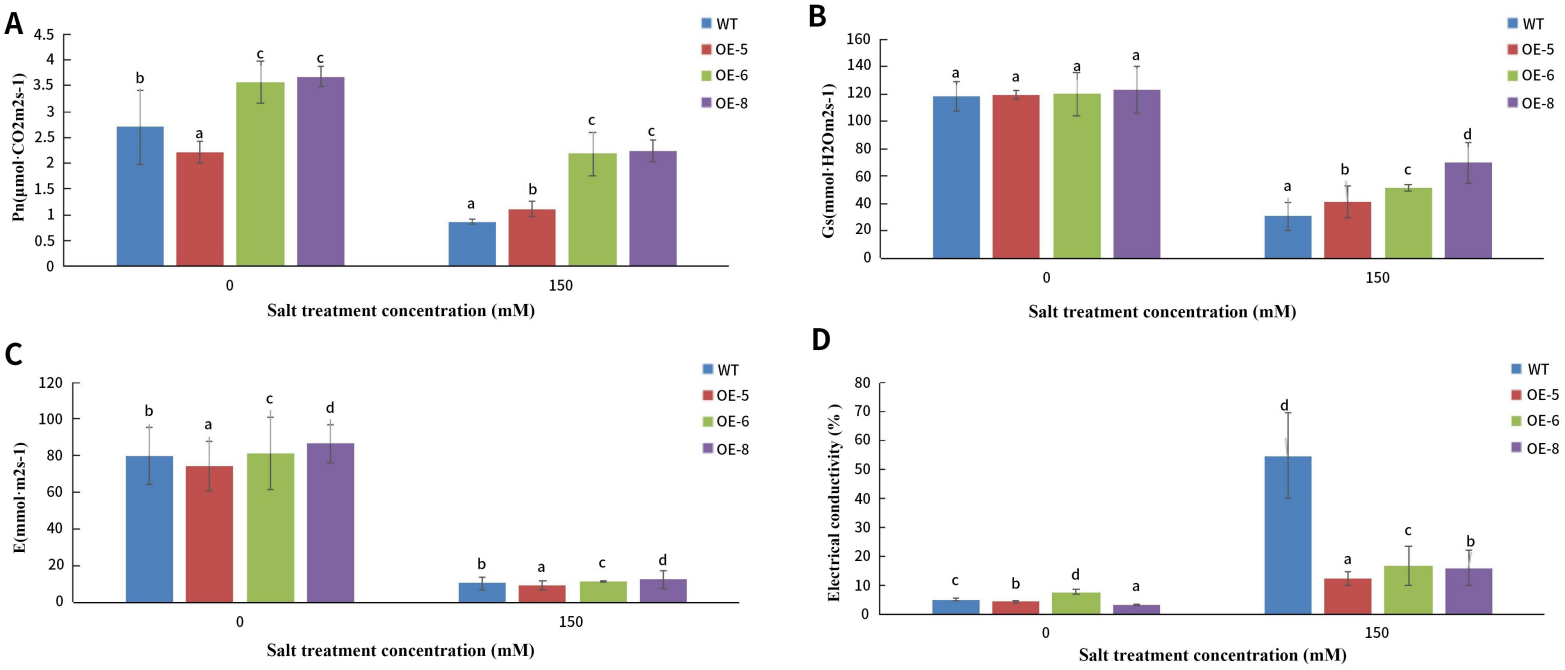
Physiological indices of *MsNAC40* over expressed plants under salt stress. **(A)** is the measurement of net photosynthetic rate of leaves; **(B)** is the measurement of stomatal conductance of leaves; **(C)** is the measurement of transpiration rate of leaves; **(D)** is the measurement of electrical conductivity of leaves.In bar charts, “abc” denotes the results of significance analysis.

As shown in **Figure 13**, the photosynthetic indexes of the three lines were significantly lower, but their conductivity was significantly higher than that of the controls after 15d of salt treatment. Based on the comprehensive analysis of the photosynthesis indexes and conductivity, the conductivity of wild-type alfalfa SY4D was significantly higher than that of the overexpression lines after salt treatment. The net photosynthetic rate, stomatal conductance and transpiration rate of the wild-type alfalfa SY4D were significantly lower than those of the overexpression lines L6 and L8, indicating that the photosynthesis of alfalfa SY4D was greatly affected by salt stress.

### 3.12 Analysis of the biochemical indicators of salt tolerance and the contents of K+ and Na+ in root and leaf tissues of alfalfa plants overexpressing the target gene

**Figure 14:**
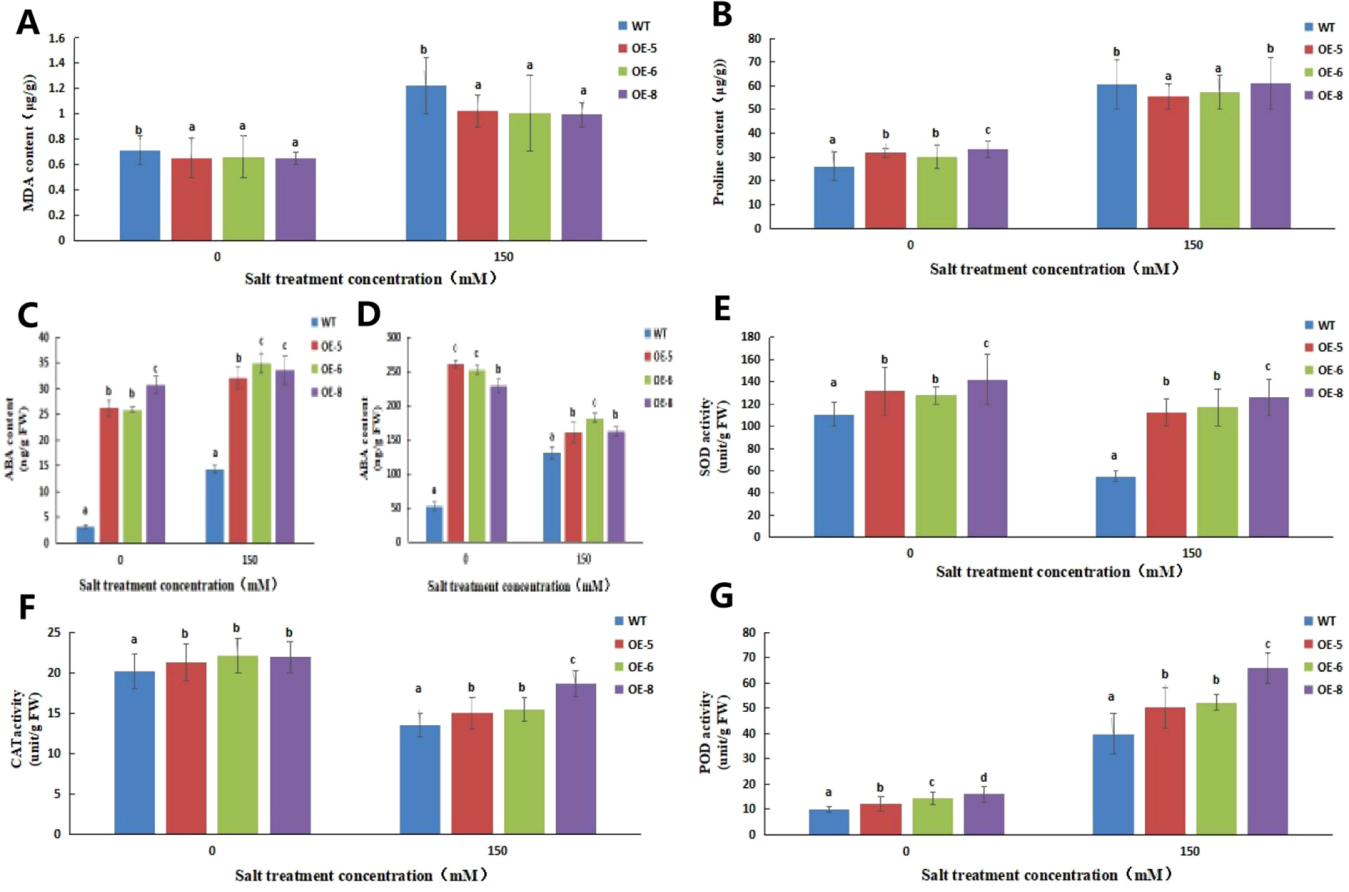
Analysis of biochemical indices of *MsNAC40* overexpressed plants under salt Stress. **(A)** is the malondialdehyde content in the leaves, **(B)** is the proline content in the leaves, **(C)** is the abscisic acid content in the roots, **(D)** is the abscisic acid content in the leaves, **(E)** is the superoxide dismutase activity, **(F)** is the catalase activity, **(G)** is the peroxidase activity.In bar charts, “abc” denotes the results of significance analysis.

The results are shown in **Figure 14** After 15d of salt treatment, the proline and malondialdehyde contents of the overexpressing lines increased significantly, but the malondialdehyde content of the control was significantly higher than that of the overexpressing lines, indicating that salt stress caused greater damage to the cell membrane of the wild type alfalfa SY4D cells. The superoxide dismutase and catalase activities decreased significantly, while that of peroxidase increased significantly in the overexpressing lines compared to the control, indicating that the antioxidant capacity of the transgenic lines was significantly improved. Abscisic acid content in the roots of both control and transgenic plants was significantly higher after 24 h of salt treatment than under normal conditions, but the abscisic acid in the roots and leaves of transgenic plants was significantly higher than that in the control. Prior to salt stress, the abscisic acid (ABA) content in the transgenic plants was significantly higher than that of the control group. After salt treatment, both transgenic and control plants showed an increase in ABA levels; however, the increase was more pronounced in the control group. While the transgenic plants also exhibited a rise in ABA levels, the magnitude of this increase was relatively smaller compared to the control group. In summary, although transgenic plants can enhance baseline ABA levels, their response to salt stress in terms of ABA accumulation is less pronounced than in the control plants.

**Figure 15:**
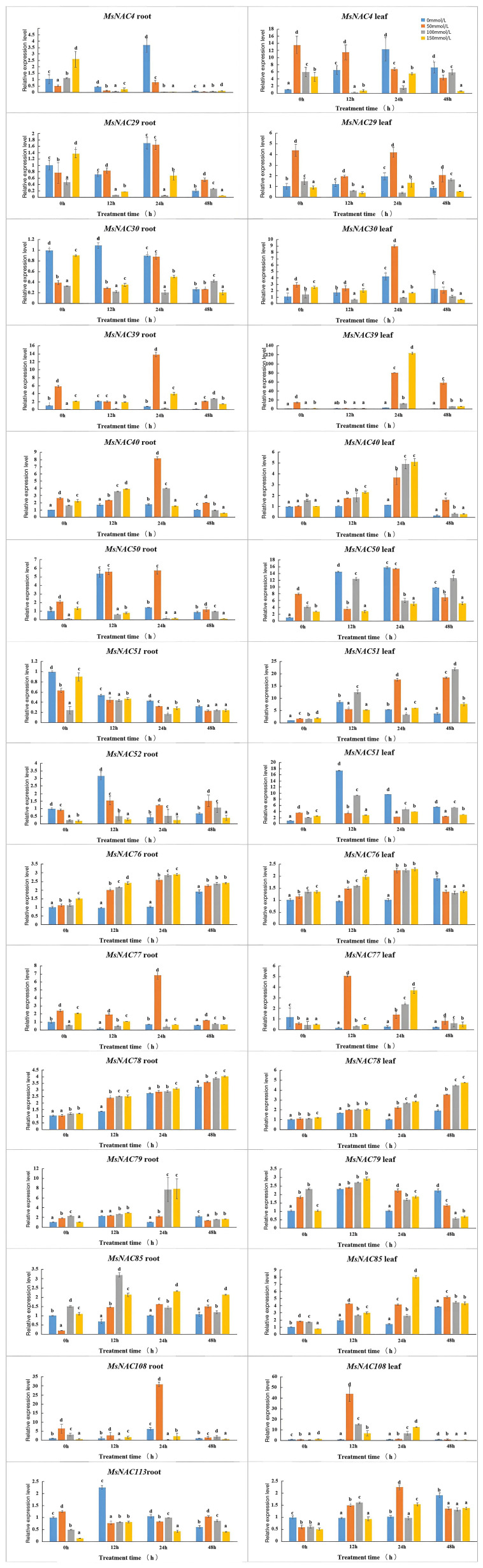
The contents of K^+^ and Na^+^ in *MsNAC40* overexpressed plants under salt stress. **(A)** is the content of Na^+^ in the root, **(B)** is the content of K^+^ in the root, **(C)** is the content of K^+^/Na^+^ in the root, **(D)** is the content of Na^+^ in the leaf, **(E)** is the content of K^+^ in the leaf, **(F)** is the content of K^+^/Na^+^ in the leaf.

As shown in **Figure 15**, the Na^+^ content in the roots and leaves of transgenic plants was significantly lower, but their K^+^/Na^+^ ratio was significantly higher under salt stress compared to control. The K^+^ content in the roots of transgenic L6 and L8 plants and the K^+^ content in the leaves of L5 and L8 plants under salt stress was significantly higher than that of the control. This indicated that the transgenic plants could effectively reduce the uptake of Na^+^ by the roots and leaves, maintain the stability of K^+^ in the root and leaf tissues, maintain the internal homeostasis of K^+^/Na^+^, and reduce the toxicity of Na^+^.

## 4 Discussion

This study conducted a comprehensive and systematic bioinformatics analysis of the alfalfa *NAC* gene members. Considering the possibility that salt-tolerant genes are also responsive to other abiotic stresses, we adopted a screening strategy with salt stress as the main stress and saline and alkaline co-stress as the secondary stress based on the transcriptomic data of alkali stress and saline and alkaline co-stress. According to the analysis of promoter cis-acting elements and salt tolerance of the subfamilies of the alfalfa *NAC* gene family, subfamily V was found to be mostly associated with stress tolerance. Moreover, based on the expression levels of the candidate genes, the *MsNAC40* gene, a member of subfamily V, was selected for subsequent analysis.

We screened 114 alfalfa *NAC* genes based on the Zhongmu No.1 genome using the Hidden Markov Model and conducted phylogenetic analyses of the alfalfa *NAC* gene family members. He et al. (2022) screened 421 alfalfa *NAC* genes from the Xinjiang Daye genome and identified 25, 42 and 47 alfalfa genes responsive to cold, drought and salt stress, respectively, via transcriptomic and qPCR analyses (He et al., 2022). In this study, phylogenetic analysis of the alfalfa NAC gene family confirmed that alfalfa is a homotetraploid. The NAC genes in the Zhongmu No.1 genome were distributed across eight chromosomes, while in the Xinjiang Large-Leaf genome, they were distributed across 32 chromosomes, reflecting the fact that the Xinjiang Large-Leaf alfalfa genome consists of four haploid genome sets, whereas Zhongmu No.1 consists of only one. Given this fundamental genomic difference, direct comparisons of NAC gene family distribution between the two genomes may have limited significance. To further advance research on the NAC gene family in alfalfa, the **Supplementary Materials** of this paper provide a comparative analysis of NAC gene members between Zhongmu No.1 and Xinjiang Large-Leaf alfalfa. The observed gene distribution aligns with patterns seen in other polyploid species, such as cotton (Wu et al., 2013), and may reflect evolutionary pressures and functional adaptations of polyploid plants under abiotic stress conditions. Finally, based on the expression levels of candidate genes, MsNAC40 from subfamily V was selected as the focus for further research.

The primary structure, tertiary structure, hydrophilicity, and prediction analysis of phosphorylation sites of the protein *MsNAC40* showed that *MsNAC40* has 329 amino acids and a molecular weight of 3.70 KDa, with the NAM a conserved structural domain, and is a hydrophilic protein with no transmembrane structure. Tissue-specific expression of *MsNAC40* was analysed, and it was found that the relative expression level of *MsNAC40* was the highest in the roots and the lowest in flowers and was expressed to different degrees in the stems, leaves and branches of alfalfa plants.

The fresh weight and plant height of L8 were significantly higher than that of the control, while the plant heights of L5 and L6 were significantly higher than those of the control. This indicated that the salt tolerance of *MsNAC40*-overexpressing plants was improved compared with that of the control. The above-ground biomass of L5 and L6 did not differ significantly from the control, probably because the duration of salt treatment was too short and *MsNAC40* didn’t play the antioxidation role in the leaves directly, resulting in the wilting and yellowing of the leaves of the overexpression plants, similar to the control leaves.

Furthermore, the photosynthetic indexes of the plant, such as net photosynthetic rate, stomatal conductance, and transpiration rate, were significantly decreased after the salt treatment compared with that before the treatment, indicating that salt stress affected the photosynthesis of the plants. The photosynthetic indexes of *MsNAC40*-overexpressing plants, except for the transpiration rate of the L5 line, were significantly higher than that of the control,It is likely that variations in expression levels among different plants contribute to the differences in net photosynthetic rate responses observed in L5 compared to L6 and L8. suggesting that the photosynthesis of *MsNAC40*-overexpressing plants was less affected by the salt stress. The conductivity of the leaves of the control group was significantly higher than that of the *MsNAC40*-overexpressing plants, indicating that the leaves of the control group were more damaged by salt stress and that the *MsNAC40*-overexpressing plants were more salt-tolerant than the control plants.

Malondialdehyde and proline contents were significantly elevated in the plant leaves after salt stress; however, the accumulation of malondialdehyde was significantly higher in the control leaves than in those of the *MsNAC40*-overexpressing plants. The proline content of the control plants did not differ significantly from that of *MsNAC40*-overexpressing L8 lines and was significantly higher in L5 and L6 plants. The variation in response to proline content between L8 and L5/L6 might be attributed to differences in expression levels across individual plants. Malondialdehyde is one of the important products of membrane lipid peroxidation, and its production can also exacerbate membrane damage (Jones, 2007), indicating that the leaves of *MsNAC40*-overexpressing plants were less damaged by salt stress. The physiological significance of proline accumulation in plants under salt stress is conflicted. One view is that proline accumulation can increase plant tolerance to osmotic stress because it can regulate the ionic balance in plants, thus maintaining the balance of intra- and extracellular concentrations and reducing cellular water loss (Wang et al., 2015). Nonetheless, proline can also protect biomolecules such as proteins and membrane lipids and enhance plant adaptation to other stresses (Ben Rejeb et al., 2012; Ghosh et al., 2022). However, judging from the significantly increased proline content in the control and overexpression plants after salt stress in this study, both control and overexpression plants were affected by salt stress, with slight differences them, suggesting that *MsNAC40* may not be associated with free proline accumulation in alfalfa.

*MsNAC40* was found to contain more cis-acting elements related to abscisic acid synthesis, and the abscisic acid content was increased in the roots and leaves of *MsNAC40*-overexpressing plants compared to the control, but the content was decreased in the leaves after 24 h of salt stress. Studies have shown that all abiotic stresses can induce a rapid increase in abscisic acid content in plants, thus increasing their stress tolerance (Dong et al., 2019). Abscisic acid induces the resynthesis of plant enzymes, increases plant salt resistance (Kou et al., 2021), significantly reduces the organellar ultrastructure damage caused by high temperatures and other adversities, and increases the stability of organelles (Lv et al., 2022; Tao et al., 2022). Thus, identifying the key genes associated with the synthesis of resistance hormones such as abscisic acid could help clarify how *MsNAC40* increases abscisic acid content in plants.

We analysed the activities of three antioxidant enzymes, superoxide dismutase, peroxide, and hydrogen peroxide, and found a significant decrease in superoxide dismutase activity after 15 d of salt treatment compared to the control treatment. This might have been because the salt treatment duration was too short, resulting in the production of other osmotic substances in the plants, thus inhibiting the large accumulation of intracellular reactive oxygen species (Anjum et al., 2015; Gill et al., 2015). The prolonged duration of salt stress increased the accumulation of reactive oxygen species, further increasing superoxide dismutase activity in the leaves (Gharsallah et al., 2016). Jin suggests that salt stress significantly increases peroxidase activity in salt-tolerant plants, thereby enhancing both salt tolerance and antioxidant responses in soybeans (Jin et al., 2019). Peroxidase activity of the *MsNAC40*-overpressing lines was significantly higher than that of the control, suggesting that the *MsNAC40*-overexpressing lines had better salt tolerance than the control. As one of the major scavengers of cellular reactive oxygen species, catalase plays an important role in plant salt tolerance (Wang et al., 2023; Zhang et al., 2013). Rout and Shaw (2001) concluded that enhanced catalase activity is closely related to plant salt tolerance (Rout and Shaw, 2001). The results of the present study revealed a significant decrease in catalase activity in the leaves of control and overexpression plants after 15 d of salt treatment compared to the normal conditions, suggesting a lower association between *MsNAC40* and catalase synthesis. However, since determining the effect of antioxidant enzyme activity only in the leaves is not comprehensive enough, enzyme activity should be further analysed in different parts of the plants under salt stress.

A balanced ratio of mineral nutrients to sodium in plants under salt-stressed environments is a physiological manifestation of plant salt tolerance, and a higher K^+^/Na^+^ ratio is one of the important indicators of plant salt tolerance (Bassil et al., 2011; Blumwald, 2000; Deinlein et al., 2014; Muchate et al., 2016; van Zelm et al., 2020) This study showed that the Na^+^ content in the roots and leaves of the *MsNAC40*-overexpressing plants was significantly lower than that of the control plants under salt stress, and the K^+^/Na^+^ was significantly higher. This indicated that *MsNAC40* could prevent Na^+^ from entering the cells, alleviate the competitive effects between K^+^ and Na^+^, promote the uptake of K^+^, reduce the ionic toxicity of Na^+^, and maintain the internal homeostasis ratio of K^+^/Na^+^ in alfalfa. Overall, the salt tolerance capacity of the *MsNAC40*-overexpressing plants was enhanced under salt stress.

## 5 Conclusions

This study identified 114 *NAC* gene family members from the Zhongmu No.1 genome for the first time and classified the genes into 13 subclasses (I to XIII). All identified genes are hydrophilic proteins. Most genes contain the conserved NAM domain, and family members generally exhibit 8 conserved motifs. All genes contain introns, with subfamilies V, VIII, and XII showing simpler structures and higher intragenic similarity. The distribution of genes across chromosomes is relatively even. The promoter regions are rich in cis-elements related to light response, hormone regulation, and abiotic stress, suggesting that the V subfamily may play a role in regulating ABA and anaerobic stress responses.

The open reading frame of the *MsNAC40* gene was 990 bp, encoding a 329 amino acids-long hydrophilic protein without a transmembrane structure, with a molecular weight of 3.70KDa and a NAM conserved structural domain. The physiological indexes of the *MsNAC40*-overexpressing plants, except conductivity, and their biochemical indexes, except malondialdehyde content, were significantly higher than in the control. Similarly, the K^+^/Na^+^ ratio in the roots and leaves of the *MsNAC40*-overexpressing plants was significantly higher than in the control under salt stress. In conclusion, the salt tolerance of *MsNAC40*-overexpressing plants was improved under salt stress compared with that of the wild-type alfalfa SY4D.

## Data Availability

The original contributions presented in the study are included in the article/**Supplementary Material**. Further inquiries can be directed to the corresponding authors.

## Supplementary material

Supplementary Figure 1 Map of overexpressed vector pCAMIBA3301.*MsNAC40.*

Supplementary Figure 2 Diagram of genetic transformation of Alfalfa. **(A)** shows the state of co.cultured leaves in the dark, **(B)** and **(C)** show the callus state in selected medium SH3a, **(D)** and **(E)** show the state of green buds emerging from callus on MSBK medium, **(E)** and **(F)** show the state of rooting of callus on SH9a medium.In bar charts, “abc” denotes the results of significance analysis.

Supplementary Figure 3 Identification of *MsNAC40* in overexpressed positive plants. M is 1500bp DNA Marker, WT is pCAMIBA3301 vector with empty plasmid as the template, and 1 to 35 are the 35 *MsNAC40*-overexpressing seedlings positive for the target gene.
