# Supplementary material for: Screening and functional characterization of salt-tolerant NAC gene family members in Medicago sativa L
Source: Front Plant Sci. 2025 Apr 1;16:1461735. doi: 10.3389/fpls.2025.1461735 (PMC11996932; doi:10.3389/fpls.2025.1461735)
Supplement: Supplementary Figure 1 — Map of overexpressed vector pCAMIBA3301.MsNAC40. [file DataSheet1.docx]

**Screening and Functional Characterization of Salt-tolerant**

**NAC gene Family Members in Medicago sativa L.**

**Supplemental files:**

**Appendix Table S1 List of Physical and chemical properties of Alfalfa *NAC* gene family members**

| Gene Name | Gene ID | Number of Amino Acid | Molecular Weight | Theoretical pI | Instability Index | Aliphatic Index | Grand Average of  Hydropathicity |
| --- | --- | --- | --- | --- | --- | --- | --- |
| *MsNAC1* | MsG0080048035.01.T01 | 358 | 40445.43 | 8.57 | 35.38 | 63.97 | -0.631 |
| *MsNAC2* | MsG0880046467.01.T07 | 383 | 43521.23 | 8.52 | 34.12 | 66.66 | -0.408 |
| *MsNAC3* | MsG0380013100.01.T01 | 306 | 35452.15 | 9.11 | 32.41 | 83.1 | -0.346 |
| *MsNAC4* | MsG0680031346.01.T01 | 236 | 27007.47 | 6.91 | 26.7 | 69.36 | -0.627 |
| *MsNAC5* | MsG0680030783.01.T01 | 315 | 36427.75 | 5.6 | 45.78 | 56 | -0.748 |
| *MsNAC6* | MsG0780040144.01.T01 | 306 | 35294.1 | 6.07 | 34.39 | 61.47 | -0.925 |
| *MsNAC7* | MsG0780036304.01.T01 | 290 | 34018.02 | 8.7 | 37.96 | 65.9 | -0.532 |
| *MsNAC8* | MsG0380015433.01.T01 | 250 | 28644.85 | 9.08 | 33.31 | 79.48 | -0.331 |
| *MsNAC9* | MsG0380015434.01.T01 | 484 | 54484.08 | 8.54 | 34.19 | 57.6 | -0.695 |
| *MsNAC10* | MsG0780036303.01.T01 | 345 | 39194.48 | 5.68 | 34.63 | 56.26 | -0.738 |
| *MsNAC11* | MsG0780036301.01.T01 | 298 | 33934.82 | 5.18 | 32.26 | 61.48 | -0.618 |
| *MsNAC12* | MsG0280010163.01.T01 | 260 | 29399.99 | 9.47 | 39.02 | 57.42 | -0.739 |
| *MsNAC13* | MsG0380017582.01.T01 | 365 | 41230.22 | 8.09 | 34.23 | 54.27 | -0.765 |
| *MsNAC14* | MsG0180000772.01.T01 | 1094 | 124666.27 | 7.97 | 34.46 | 75.61 | -0.51 |
| *MsNAC15* | MsG0380017974.01.T01 | 376 | 43006.34 | 6.57 | 42.33 | 62.79 | -0.687 |
| *MsNAC16* | MsG0180003817.01.T01 | 280 | 32339.56 | 5.89 | 33.18 | 66.14 | -0.568 |
| *MsNAC17* | MsG0780040754.01.T01 | 291 | 33546.68 | 5.43 | 39.62 | 69.31 | -0.642 |
| *MsNAC18* | MsG0280009717.01.T01 | 314 | 36172.1 | 8.31 | 41.27 | 63.03 | -0.617 |

| Gene Name | Gene ID | Number of Amino Acid | Molecular Weight | Theoretical pI | Instability Index | Aliphatic Index | Grand Average of  Hydropathicity |
| --- | --- | --- | --- | --- | --- | --- | --- |
| *MsNAC19* | MsG0880044712.01.T01 | 398 | 44930.75 | 8.25 | 42.01 | 68.84 | -0.542 |
| *MsNAC20* | MsG0580026147.01.T01 | 405 | 46353.34 | 8.06 | 37.5 | 70.27 | -0.589 |
| *MsNAC21* | MsG0680030755.01.T01 | 377 | 43030.42 | 6.54 | 43.91 | 65.44 | -0.647 |
| *MsNAC22* | MsG0880047771.01.T01 | 97 | 11802.73 | 9.75 | 45.7 | 78.35 | -0.5 |
| *MsNAC23* | MsG0580029691.01.T01 | 295 | 34217.44 | 8.43 | 28.63 | 65.02 | -0.787 |
| *MsNAC24* | MsG0280009641.01.T01 | 310 | 35773.96 | 6.8 | 29.49 | 63.48 | -0.812 |
| *MsNAC25* | MsG0580027721.01.T01 | 311 | 35910.94 | 6.15 | 31.71 | 61.7 | -0.845 |
| *MsNAC26* | MsG0280009650.01.T01 | 262 | 30145.28 | 6.8 | 30.33 | 57.63 | -0.979 |
| *MsNAC27* | MsG0280009642.01.T01 | 298 | 34462.56 | 8.2 | 36.37 | 65.77 | -0.816 |
| *MsNAC28* | MsG0580027720.01.T01 | 671 | 76478.94 | 9.26 | 42.75 | 78.3 | -0.594 |
| *MsNAC29* | MsG0380015185.01.T01 | 688 | 78673.86 | 5.86 | 36.92 | 72.95 | -0.555 |
| *MsNAC30* | MsG0580028885.01.T01 | 651 | 74935.83 | 5.94 | 43 | 68.1 | -0.595 |
| *MsNAC31* | MsG0280009479.01.T01 | 347 | 40027.07 | 5.14 | 46.17 | 59.57 | -0.723 |
| *MsNAC32* | MsG0380013184.01.T01 | 561 | 62465.52 | 4.52 | 37.56 | 71.66 | -0.402 |
| *MsNAC33* | MsG0480022079.01.T01 | 115 | 13578.36 | 5.34 | 36.01 | 71.22 | -0.62 |
| *MsNAC34* | MsG0580026213.01.T01 | 273 | 31644.61 | 6.91 | 30.42 | 59.63 | -0.779 |
| *MsNAC35* | MsG0280010890.01.T01 | 137 | 15868.95 | 9.28 | 46.83 | 66.93 | -0.729 |
| *MsNAC36* | MsG0380016874.01.T01 | 292 | 33795.08 | 6.09 | 36.01 | 61.4 | -0.835 |
| *MsNAC37* | MsG0480023497.01.T01 | 287 | 33112.27 | 5.85 | 34.63 | 60.17 | -0.808 |
| *MsNAC38* | MsG0380016358.01.T01 | 215 | 24730.78 | 8.3 | 46.27 | 53.16 | -0.857 |
| *MsNAC39* | MsG0280010252.01.T08 | 151 | 17748.58 | 9.34 | 28.03 | 78.74 | -0.223 |
| *MsNAC40* | MsG0880044354.01.T01 | 329 | 40359.64 | 7.23 | 40.14 | 68.52 | -0.504 |
| *MsNAC41* | MsG0780040143.01.T01 | 121 | 13662.51 | 9.45 | 29.34 | 69.34 | -0.559 |

| Gene Name | Gene ID | Number of Amino Acid | Molecular Weight | Theoretical pI | Instability Index | Aliphatic Index | Grand Average of  Hydropathicity |
| --- | --- | --- | --- | --- | --- | --- | --- |
| *MsNAC42* | MsG0280010253.01.T01 | 192 | 21533.39 | 9.76 | 33.72 | 55.94 | -0.801 |
| *MsNAC43* | MsG0480022423.01.T01 | 347 | 38890.93 | 7.75 | 44.43 | 53.43 | -0.898 |
| *MsNAC44* | MsG0480022472.01.T01 | 196 | 21339.99 | 9.2 | 49.63 | 64.69 | -0.526 |
| *MsNAC45* | MsG0580024680.01.T01 | 348 | 39293.59 | 5.43 | 51.27 | 54.63 | -0.673 |
| *MsNAC46* | MsG0880046050.01.T01 | 319 | 36447.7 | 5.31 | 44.43 | 63.23 | -0.603 |
| *MsNAC47* | MsG0880045010.01.T01 | 573 | 62955.26 | 4.67 | 49.16 | 59.41 | -0.56 |
| *MsNAC48* | MsG0480023461.01.T01 | 490 | 53937.63 | 4.09 | 44.91 | 64.88 | -0.537 |
| *MsNAC49* | MsG0380016826.01.T01 | 739 | 83631.96 | 5.17 | 41.94 | 86.64 | -0.404 |
| *MsNAC50* | MsG0480021763.01.T01 | 230 | 26142.82 | 9.22 | 44.48 | 72.48 | -0.649 |
| *MsNAC51* | MsG0280010527.01.T01 | 254 | 28768.97 | 5.84 | 50.59 | 61.38 | -0.81 |
| *MsNAC52* | MsG0480020508.01.T01 | 261 | 29881.08 | 5.7 | 48.42 | 57.47 | -0.774 |
| *MsNAC53* | MsG0280006839.01.T01 | 196 | 22520.09 | 8.39 | 51.04 | 54.69 | -0.93 |
| *MsNAC54* | MsG0780041150.01.T01 | 196 | 22590.18 | 7.7 | 48.28 | 54.69 | -0.933 |
| *MsNAC55* | MsG0180001851.01.T01 | 329 | 37546.19 | 5.72 | 50.14 | 77.63 | -0.733 |
| *MsNAC56* | MsG0180001853.01.T01 | 319 | 36709.49 | 5.48 | 48.11 | 81.88 | -0.667 |
| *MsNAC57* | MsG0180002956.01.T01 | 331 | 38202.54 | 4.84 | 42.91 | 74.83 | -0.822 |
| *MsNAC58* | MsG0780041697.01.T01 | 580 | 66195.03 | 6.53 | 30.95 | 84.69 | -0.586 |
| *MsNAC59* | MsG0180000238.01.T01 | 338 | 38503.05 | 6.1 | 43.8 | 76.69 | -0.776 |
| *MsNAC60* | MsG0480020922.01.T01 | 92 | 10854.43 | 9.3 | 47.36 | 79.46 | -0.335 |
| *MsNAC61* | MsG0180001156.01.T01 | 222 | 24702.87 | 5.22 | 35.42 | 78.65 | -0.253 |
| *MsNAC62* | MsG0180001296.01.T01 | 247 | 27554.64 | 4.67 | 36.4 | 61.7 | -0.416 |
| *MsNAC63* | MsG0180001295.01.T01 | 246 | 27607.67 | 4.38 | 29.63 | 68.98 | -0.288 |
| *MsNAC64* | MsG0680033115.01.T01 | 179 | 19969.47 | 4.44 | 32.81 | 70.78 | -0.232 |

| Gene Name | Gene ID | Number of Amino Acid | Molecular Weight | Theoretical pI | Instability Index | Aliphatic Index | Grand Average of  Hydropathicity |
| --- | --- | --- | --- | --- | --- | --- | --- |
| *MsNAC65* | MsG0680033120.01.T01 | 238 | 26446.74 | 4.58 | 29.97 | 77.48 | -0.268 |
| *MsNAC66* | MsG0480022691.01.T01 | 192 | 22303.69 | 4.82 | 50.21 | 62.34 | -0.831 |
| *MsNAC67* | MsG0380016844.01.T01 | 206 | 23520.13 | 4.24 | 38.96 | 76.55 | -0.417 |
| *MsNAC68* | MsG0480023468.01.T01 | 178 | 20480.8 | 4.77 | 47.28 | 70.56 | -0.594 |
| *MsNAC69* | MsG0180005331.01.T01 | 595 | 67528.04 | 4.6 | 49.5 | 68.92 | -0.527 |
| *MsNAC70* | MsG0780035923.01.T01 | 248 | 27863.01 | 8.63 | 40.26 | 54.6 | -0.827 |
| *MsNAC71* | MsG0680034895.01.T01 | 351 | 40445.88 | 8.46 | 36.54 | 67.95 | -0.517 |
| *MsNAC72* | MsG0580030106.01.T01 | 206 | 23579.76 | 9.72 | 32.78 | 65.19 | -0.735 |
| *MsNAC73* | MsG0580030105.01.T01 | 222 | 25423.68 | 9.16 | 28.26 | 56.17 | -0.813 |
| *MsNAC74* | MsG0580030108.01.T01 | 394 | 44741.15 | 8.93 | 37.16 | 70 | -0.457 |
| *MsNAC75* | MsG0680035337.01.T01 | 88 | 10352.02 | 6.11 | 28.34 | 79.66 | -0.303 |
| *MsNAC76* | MsG0480023575.01.T01 | 116 | 13575.82 | 9.59 | 45.7 | 78.88 | -0.336 |
| *MsNAC77* | MsG0380016978.01.T01 | 284 | 32582.64 | 7.57 | 44.14 | 51.76 | -0.681 |
| *MsNAC78* | MsG0280009432.01.T01 | 339 | 39064.47 | 5.64 | 50.14 | 66.46 | -0.838 |
| *MsNAC79* | MsG0080048648.01.T01 | 355 | 41189.85 | 6.89 | 42.09 | 51.61 | - 1.048 |
| *MsNAC80* | MsG0480019602.01.T01 | 354 | 41043.7 | 6.89 | 41.09 | 52.57 | - 1.044 |
| *MsNAC81* | MsG0880042925.01.T01 | 430 | 48423.46 | 5.83 | 42.52 | 59.42 | -0.81 |
| *MsNAC82* | MsG0880042902.01.T01 | 429 | 48308.33 | 5.83 | 43.04 | 59.56 | -0.811 |
| *MsNAC83* | MsG0880046147.01.T01 | 152 | 17989.24 | 9.24 | 27.98 | 55.13 | -0.988 |
| *MsNAC84* | MsG0580024492.01.T01 | 344 | 39868.27 | 6.27 | 51.46 | 60.38 | -0.929 |
| *MsNAC85* | MsG0180005288.01.T01 | 298 | 34587.31 | 5.48 | 45.53 | 63.49 | -0.84 |
| *MsNAC86* | MsG0480019665.01.T01 | 64 | 7236.5 | 9.84 | 21.68 | 73.28 | -0.322 |
| *MsNAC87* | MsG0580025064.01.T01 | 352 | 40618.49 | 5.2 | 54.62 | 68.41 | -0.738 |

| Gene Name | Gene ID | Number of Amino Acid | Molecular Weight | Theoretical pI | Instability Index | Aliphatic Index | Grand Average of  Hydropathicity |
| --- | --- | --- | --- | --- | --- | --- | --- |
| *MsNAC88* | MsG0880045659.01.T01 | 359 | 42030.6 | 6.4 | 52.97 | 66.55 | -0.748 |
| *MsNAC89* | MsG0880042867.01.T01 | 163 | 19225.19 | 9.12 | 34.54 | 79.45 | -0.533 |
| *MsNAC90* | MsG0880042870.01.T01 | 662 | 77042.21 | 9.25 | 40.32 | 89.88 | -0.383 |
| *MsNAC91* | MsG0880042871.01.T01 | 469 | 54569.29 | 5.33 | 45.12 | 68.32 | -0.61 |
| *MsNAC92* | MsG0880042865.01.T01 | 338 | 38658.14 | 5.9 | 57.29 | 54.23 | -0.717 |
| *MsNAC93* | MsG0880042866.01.T01 | 413 | 46914.17 | 5.02 | 49.96 | 67.24 | -0.661 |
| *MsNAC94* | MsG0880042862.01.T01 | 514 | 58037.69 | 4.7 | 55.47 | 73.74 | -0.535 |
| *MsNAC95* | MsG0380016673.01.T01 | 826 | 92371.22 | 5.5 | 44.35 | 83.67 | -0.414 |
| *MsNAC96* | MsG0380016675.01.T01 | 621 | 69776.38 | 5.68 | 40.56 | 74.24 | -0.48 |
| *MsNAC97* | MsG0580028114.01.T01 | 638 | 71128.19 | 4.87 | 50.92 | 69.87 | -0.53 |
| *MsNAC98* | MsG0880047087.01.T01 | 308 | 35014.24 | 5.01 | 52.26 | 69.38 | -0.642 |
| *MsNAC99* | MsG0780040001.01.T01 | 125 | 14849.91 | 9.2 | 50.19 | 64.8 | -0.585 |
| *MsNAC100* | MsG0780040000.01.T01 | 178 | 20759.61 | 9.12 | 49.82 | 64.1 | -0.631 |
| *MsNAC101* | MsG0780039999.01.T01 | 196 | 23025.27 | 8.9 | 33.75 | 63.16 | -0.621 |
| *MsNAC102* | MsG0780040004.01.T01 | 155 | 18684.27 | 6.15 | 29.87 | 79.94 | -0.412 |
| *MsNAC103* | MsG0680034839.01.T01 | 210 | 24686.98 | 4.52 | 62.88 | 70.1 | -0.624 |
| *MsNAC104* | MsG0280006777.01.T01 | 475 | 53495.22 | 5.89 | 37.88 | 82.04 | -0.473 |
| *MsNAC105* | MsG0480022322.01.T01 | 319 | 35999.19 | 5.81 | 39.58 | 67.77 | -0.792 |
| *MsNAC106* | MsG0280010788.01.T01 | 286 | 32566.73 | 8.72 | 36.21 | 63.32 | -0.81 |
| *MsNAC107* | MsG0480021923.01.T01 | 284 | 32049.94 | 8.93 | 37.66 | 64.44 | -0.86 |
| *MsNAC108* | MsG0480023787.01.T01 | 351 | 39622.01 | 7.69 | 50 | 55.24 | - 1.012 |
| *MsNAC109* | MsG0780037452.01.T01 | 540 | 60869.64 | 5.01 | 50.38 | 69.94 | -0.6 |
| *MsNAC110* | MsG0580027097.01.T01 | 457 | 51536.24 | 4.94 | 50.16 | 66.52 | -0.808 |

| Gene Name | Gene ID | Number of Amino Acid | Molecular Weight | Theoretical pI | Instability Index | Aliphatic Index | Grand Average of  Hydropathicity |
| --- | --- | --- | --- | --- | --- | --- | --- |
| *MsNAC111* | MsG0180005097.01.T01 | 411 | 46628.47 | 5.41 | 51.1 | 75.4 | -0.562 |
| *MsNAC112* | MsG0180005167.01.T01 | 306 | 35196.92 | 5.68 | 53.14 | 79.28 | -0.529 |
| *MsNAC113* | MsG0180005098.01.T01 | 296 | 33966.15 | 5.84 | 55.71 | 69.76 | -0.858 |
| *MsNAC114* | MsG0180005166.01.T01 | 342 | 38749.46 | 5.28 | 54.03 | 74.39 | -0.807 |

**Appendix Table S2 Partial correspondence between Xinjiang Daye and Zhongmu No.1 in**

***NAC* gene family**

| Xinjiang Daye Gene ID | Zhongmu No.1 Gene ID | Zhongmu No.1 Gene Name |
| --- | --- | --- |
| *MS.gene044393.t1* | *MsG0080048035.01.T01* | *MsNAC1* |
| *MS.gene03529.t1* | *MsG0380013100.01.T01* | *MsNAC3* |
| *MS.gene042585.t1* | *MsG0680031346.01.T01* | *MsNAC4* |
| *MS.gene031060.t1* | *MsG0680030783.01.T01* | *MsNAC5* |
| *MS.gene51935.t1* | *MsG0780040144.01.T01* | *MsNAC6* |
| *MS.gene98838.t1* | *MsG0380015433.01.T01* | *MsNAC8* |
| *MS.gene073051.t1* | *MsG0280010163.01.T01* | *MsNAC12* |
| *MS.gene021500.t1* | *MsG0380017582.01.T01* | *MsNAC13* |
| *MS.gene91196.t1* | *MsG0180003817.01.T01* | *MsNAC16* |
| *MS.gene96987.t1* | *MsG0780040754.01.T01* | *MsNAC17* |
| *MS.gene77666.t1* | *MsG0280009717.01.T01* | *MsNAC18* |
| *MS.gene031017.t1* | *MsG0680030755.01.T01* | *MsNAC21* |
| *MS.gene049696.t1* | *MsG0380015185.01.T01* | *MsNAC29* |
| *MS.gene063613.t1* | *MsG0580028885.01.T01* | *MsNAC30* |
| *MS.gene50991.t1* | *MsG0380013184.01.T01* | *MsNAC32* |
| *MS.gene68424.t1* | *MsG0380013184.01.T01* | *MsNAC32* |
| *MS.gene51895.t1* | *MsG0580026213.01.T01* | *MsNAC34* |
| *MS.gene065170.t1* | *MsG0380016874.01.T01* | *MsNAC36* |
| *MS.gene51895.t1* | *MsG0480023497.01.T01* | *MsNAC37* |
| *MS.gene032315.t1* | *MsG0880044354.01.T01* | *MsNAC40* |
| *MS.gene031383.t1* | *MsG0280010253.01.T01* | *MsNAC42* |
| *MS.gene39413.t1* | *MsG0480022423.01.T01* | *MsNAC43* |
| *MS.gene09659.t1* | *MsG0580024680.01.T01* | *MsNAC45* |
| *MS.gene46348.t1* | *MsG0880046050.01.T01* | *MsNAC46* |
| *MS.gene052297.t1* | *MsG0480021763.01.T01* | *MsNAC50* |
| *MS.gene35694.t1* | *MsG0280010527.01.T01* | *MsNAC51* |
| *MS.gene03868.t1* | *MsG0480020508.01.T01* | *MsNAC52* |
| *MS.gene24711.t1* | *MsG0280006839.01.T01* | *MsNAC53* |
| *MS.gene073916.t1* | *MsG0180001851.01.T01* | *MsNAC55* |
| *MS.gene90002.t1* | *MsG0180002956.01.T01* | *MsNAC57* |
| *MS.gene22857.t1* | *MsG0180000238.01.T01* | *MsNAC59* |
| *MS.gene051332.t1* | *MsG0480022691.01.T01* | *MsNAC66* |
| *MS.gene013303.t1* | *MsG0380016844.01.T01* | *MsNAC67* |
| *MS.gene28382.t1* | *MsG0480023468.01.T01* | *MsNAC68* |
| *MS.gene33909.t1* | *MsG0180005331.01.T01* | *MsNAC69* |
| *MS.gene05203.t1* | *MsG0680034895.01.T01* | *MsNAC71* |

| Xinjiang Daye Gene ID | Zhongmu No.1 Gene ID | Zhongmu No.1 Gene Name |
| --- | --- | --- |
| *MS.gene013174.t1* | *MsG0380016978.01.T01* | *MsNAC77* |
| *MS.gene60913.t1* | *MsG0280009432.01.T01* | *MsNAC78* |
| *MS.gene75752.t1* | *MsG0080048648.01.T01* | *MsNAC79* |
| *MS.gene067326.t1* | *MsG0880042925.01.T01* | *MsNAC81* |
| *MS.gene006043.t1* | *MsG0180005288.01.T01* | *MsNAC85* |
| *MS.gene050978.t1* | *MsG0880042867.01.T01* | *MsNAC89* |
| *MS.gene81073.t1* | *MsG0880042871.01.T01* | *MsNAC91* |
| *MS.gene08493.t1* | *MsG0880042865.01.T01* | *MsNAC92* |
| *MS.gene81071.t1* | *MsG0880042866.01.T01* | *MsNAC93* |
| *.MS.gene066274.t1* | *MsG0880042862.01.T01* | *MsNAC94* |
| *MS.gene013480.t1* | *MsG0380016675.01.T01* | *MsNAC96* |
| *MS.gene009957.t1* | *MsG0580028114.01.T01* | *MsNAC97* |
| *MS.gene84297.t1* | *MsG0880047087.01.T01* | *MsNAC98* |
| *MS.gene54981.t1* | *MsG0780040000.01.T01* | *MsNAC100* |
| *MS.gene033668.t1* | *MsG0780039999.01.T01* | *MsNAC101* |
| *MS.gene033670.t1* | *MsG0780040004.01.T01* | *MsNAC102* |
| *MS.gene047365.t1* | *MsG0280006777.01.T01* | *MsNAC104* |
| *MS.gene26389.t1* | *MsG0480022322.01.T01* | *MsNAC105* |
| *MS.gene31423.t1* | *MsG0280010788.01.T01* | *MsNAC106* |
| *MS.gene006632.t1* | *MsG0480021923.01.T01* | *MsNAC107* |
| *MS.gene021500.t1* | *MsG0480023787.01.T01* | *MsNAC108* |
| *MS.gene035107.t1* | *MsG0780037452.01.T01* | *MsNAC109* |
| *MS.gene78720.t1* | *MsG0580027097.01.T01* | *MsNAC110* |
| *MS.gene34123.t1* | *MsG0180005097.01.T01* | *MsNAC111* |
| *MS.gene005874.t1* | *MsG0180005166.01.T01* | *MsNAC114* |

**Plant materials and cultivation conditions**

The experimental variety of alfalfa used in this study was bred by the Institute of Animal Husbandry, Chinese Academy of Agricultural Sciences. The plant material used for the experiment consisted of four-week-old single-node cuttings of Zhongmu No. 1. These were cultured in an artificial climate incubator with a photoperiod of 16 hours light and 8 hours dark, at day and night temperatures of 26°C and 24°C, respectively. The plants were grown in 1/2 Hoagland nutrient solution (30 × 20 cm, plastic containers). The experimental materials were divided into six groups, with three biological replicates per group.

Table 4 List of Formula of Hoagland nutrient solution

| Macromineral | Concentration（mM） | Mmicroelement | Concentration（mM） |
| --- | --- | --- | --- |
| Ca（NO₃) 2·4H₂O | 4 | MnSO4·4H₂O | 9.5×10-3 |
| KNO₃ | 4 | CuSO4·5H₂O | 3×10-4 |
| MgSO4·7H₂O | 2 | ZnSO4·7H₂O | 1×10-3 |
| NH₄H₂PO₄ | 1 | H₃BO₃  （NH4 ）6Mo₇O24·4H₂O  Fe-EDTA | 1.5×10-2  3×10-4  0.2 |

For the study, RNA was extracted from the second and third leaves at the stem tip and the 3 cm root tip of the four-week-old hydroponically grown Medicago 1 seedlings treated with salt for 0 h, 12 h, 24 h, and 48 h, as well as from the seedlings cultured in ddH2O for the same durations. The extracted RNA was stored in an ultra-low temperature freezer at -80℃. The RNA was then diluted to the same concentration, and cDNA was synthesized using the same concentration of RNA as a template. The RNA extraction method followed the instructions provided by Vazyme for their reverse transcriptase. Real-time fluorescence quantitative reaction systems were prepared, and the real-time fluorescence quantitative reaction conditions referred to the instructions for the qPCR enzyme produced by Vazyme.

**Vector Construction**

Using the Vazyme RNA-rc401 kit, RNA was extracted from the young leaves of 4-week-old alfalfa (Medicago sativa) SY4D. cDNA was synthesized from the extracted RNA using the HiScript® III RT SuperMix for qPCR (+gDNA wiper) from Novozan. Primers for MsNAC40-F/R were designed using Primer 5.0, and the cDNA from Medicago sativa SY4D was used as the template. PCR amplification was performed using Novozan's 2 × Phanta® Flash Master Mix to obtain the MsNAC40 gene sequence. The 3301MsNAC40-F/R primers were designed with Nco1 and Pml1 enzymatic cleavage sites (Table 3) to obtain the target gene fragment with homologous arms for ligation into the linear vector pCAMIBA3301 digested with Nco1 and Pml1 restriction enzymes. Colony PCR of the transformed E. coli cells detected no error, and the E. coli cells were transferred into Agrobacterium EHA105.

Using SnapGene software to construct the overexpression vector map of MsNAC40 in pCAMIBA3301. The vector map is as follows:
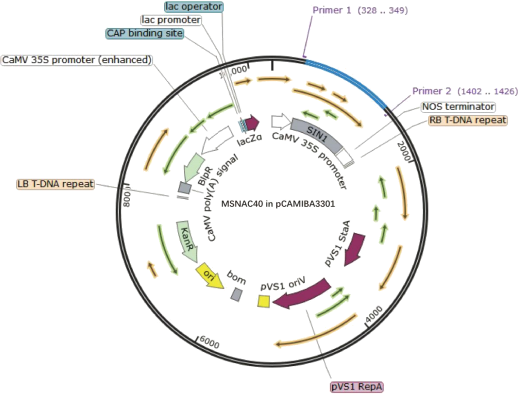


**Figure S1 Map of overexpressed vector pCAMIBA3301-*MsNAC40***

**Detailed Experimental Procedures for Genetic Transformation of Alfalfa**

The vector was constructed and transformed into Agrobacterium EHA105 cells, which were then used for the leaf disc transformation of young leaves of 4-weeks-old alfalfa SY4D.

The constructed plasmid vector was transformed into Agrobacterium tumefaciens strain EHA105, and the culture was incubated on a shaker/incubator at 200 rpm and 28°C for approximately two days, until the culture reached an OD600 of 0.8. The Agrobacterium suspension was centrifuged at 5000 rpm for 10 minutes to pellet the bacteria, which were then resuspended in infiltration medium to an OD600 of 0.4.Four-week-old young leaves of Medicago sativa SY4D were surface-sterilized by washing with 75% ethanol for 15 seconds, followed by rinsing with double-distilled water (ddH2O) for 1 minute, then disinfected with 15% sodium hypochlorite for 8 minutes and washed three times with ddH2O. The sterilized leaves were placed in sterile culture bottles and infiltrated with the Agrobacterium suspension. The culture bottles were placed in a polycarbonate desiccator connected to a vacuum pump to evacuate the air for 10 minutes, then placed in an ultrasonic cleaner for 15 minutes, and again in the desiccator for another 10 minutes under vacuum. The treated leaves were dried on sterile filter paper.

The transformed leaves were co-cultured in SH3a media for 20 h in the dark and then transferred into the selection medium . The formed calluses were cultured in the dark for 2 to 3 months and were transferred to the MSBK medium for about one week under the photoperiod cycle of 16 h light/8 h dark for 30 to 45 d. The green-sprouting calluses were transferred to SH9a medium until the tissues regenerated into plantlets. The regenerated plantlets were then grown in the glasshouse.


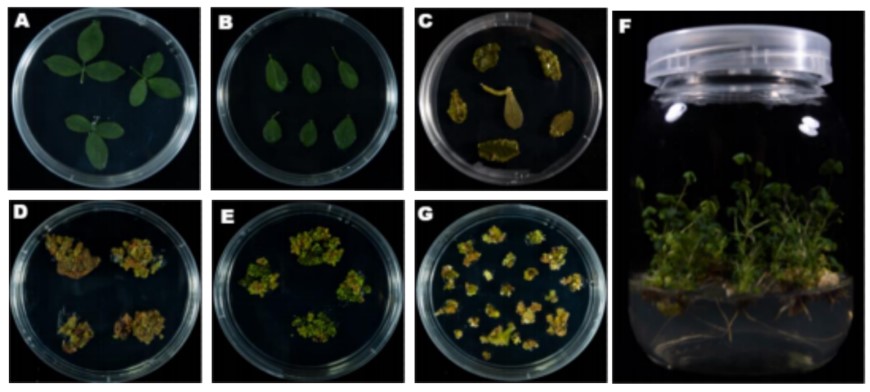


**Figure.S2 Diagram of genetic transformation of Alfalfa**

Note: A shows the state of co-cultured leaves in the dark, B and C show the callus state in selected medium SH3a, D and E show the state of green buds emerging from callus on MSBK medium, E and F show the state of rooting of callus on SH9a medium.

**Identification of MsNAC40 Overexpressing Positive Alfalfa Plants**

As depicted in Fig S3, through the operation of Agrobacterium leaf disc transformation, we found that 35 MsNAC40-overexpressing alfalfa seedlings were positive for the target gene (Figure S3.A). The first batch of alfalfa seedlings used for the identification of positive plants contained 1 to 7 lines, and sequencing results showed that the amplified gene from the seven lines was consistent with the target sequence (Figure S3.B), similar to those amplified from lines 8 to 35 (Figure S3.C).


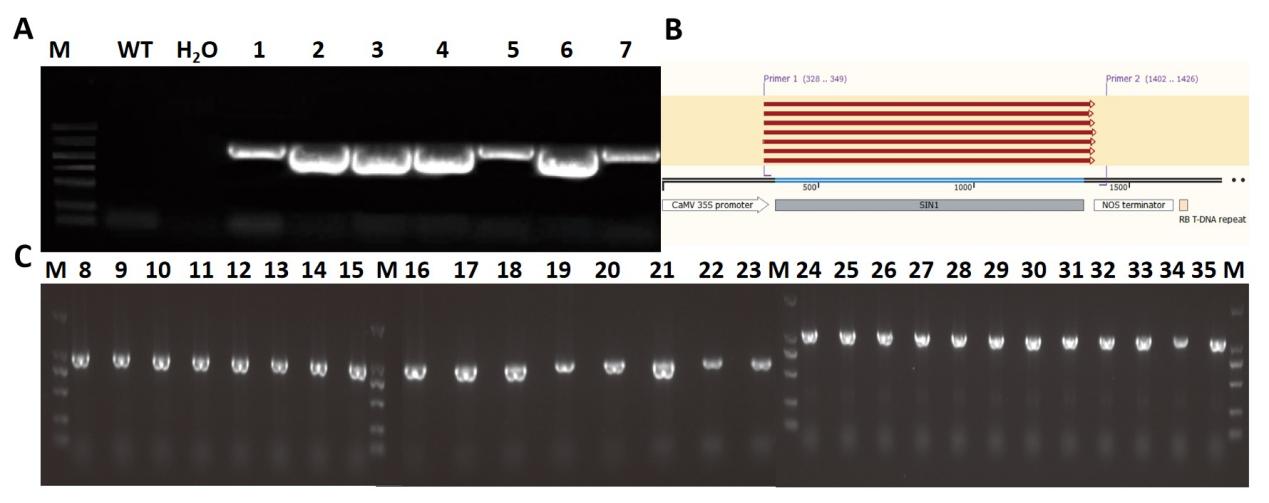


**Figure S3 Expression level of *MsNAC40* in overexpressed positive plants**

M is 1500bp DNA Marker, WT is pCAMIBA3301 vector with empty plasmid as the template, and 1 to 35 are the 35 MsNAC40.overexpressing seedlings positive for the target gene.

**Genetic Transformation of Alfalfa (Medicago sativa)**

The media required for callus culture of alfalfa include: infection medium, co-cultivation medium, selection medium, regeneration medium, shoot induction medium, and rooting medium. The formulation of each medium is provided below for a volume of 1 liter, with pH adjusted to 5.8.

Infection Medium: SH medium (14.21g), Sucrose (20g), 2,4-D (1mg/mL, 4mL), BAP (1mg/mL, 0.5mL)

Co-cultivation Medium: SH medium (14.21g), Sucrose (20g), Phytagel (3.2g), 2,4-D (1mg/mL, 4mL), BAP (1mg/mL, 0.5mL)

Selection Medium: SH medium (14.21g), Sucrose (20g), Phytagel (3.2g), 2,4-D (1mg/mL, 4mL), BAP (1mg/mL, 0.5mL), Cefotaxime (200mg), Timentin (200mg), PPT (1mg)

Regeneration Medium: MS medium (4.43g), Sucrose (30g), Phytagel (3.2g), BAP (1mg/mL, 0.5mL), Kinetin (1mg/mL, 1mL), Cefotaxime (200mg), Timentin (200mg), PPT (0.5mg)

Shoot Induction Medium: SH medium (14.21g), Sucrose (10g), Phytagel (3.2g), Cefotaxime (200mg), Timentin (200mg), PPT (0.5mg)

Rooting Medium: MS medium (4.43g), Sucrose (8g), Agar (7.5g), Cefotaxime (200mg), Timentin (200mg), PPT (0.5mg)
